# Strengthening the community support group to improve maternal and neonatal health seeking behaviors: A cluster-randomized controlled trial in Satkhira District, Bangladesh

**DOI:** 10.1371/journal.pone.0212847

**Published:** 2019-02-28

**Authors:** Ruoyan Gai Tobe, Mohammad Tajul Islam, Yukie Yoshimura, Jahangir Hossain

**Affiliations:** 1 Department of Health Policy, National Center for Child Health and Development, Tokyo, Japan; 2 MaMoni HSS Project, Save the Children International, Dhaka, Bangladesh; 3 SHASTO Project, JICA Bangladesh, Dhaka, Bangladesh; 4 CARE Bangladesh, Dhaka, Bangladesh; TNO, NETHERLANDS

## Abstract

**Background:**

Although achieved development goals on maternal and child health, in the era of Sustainable Development Goals (SDGs), Bangladesh still needs to promote skilled attendance at birth as well as a continuum of care for mothers and babies. How to implement effective interventions by strengthening the community health system also remains as a crucial policy issue. The objective of the proposed study is to evaluate the impact of a community-based intervention as part of a bilateral development aid project on utilization of maternal and neonatal care provided by skilled providers and qualified facilities.

**Methods:**

A cluster randomized trial was conducted in Kalaroa Upazila of Satkhira District. Community Clinics (CCs) in the study setting were randomly allocated to either intervention or control. We recruited all eligible women covered by CC catchment areas who gave a birth during the past 12 months of data collection at the baseline and end-line surveys. In the intervention areas, three Community Support Groups (CSGs) were developed in each of the CC areas. The members of CSG were trained to identify pregnant women, educate community people on pregnancy related danger signs, and encourage them for utilization of skilled services in the community and health facilities. The primary outcomes were the utilization of services for antenatal care, delivery, postnatal care and sick newborns. Difference-in-Difference (DID) analysis was performed to identify the changes by the intervention with adjustment of cluster effects by generalized mixed effects regression models.

**Result:**

The major indicators of the utilization of maternal and neonatal care among pregnant women with different wealth status showed significant improvement after the intervention. The impacts of the intervention were in particular significant among the women of 2^nd^ and 3^rd^ quintiles of household wealth status. The use of CCs increased after the intervention and private hospitals / clinics served as the major health providers. The study also identified increased practices of cesarean section.

**Conclusion:**

The success of the intervention suggests a potential of the government efforts to strengthen the community support system for promotion of safe motherhood. The intervention helps to identify and remove existing and emerging barriers that lie between women and healthcare providers for safe motherhood and continuum of care.

**Trial registration:**

UMIN Clinical Trial Registry UMIN000031789.

## Introduction

**Fig 1:**
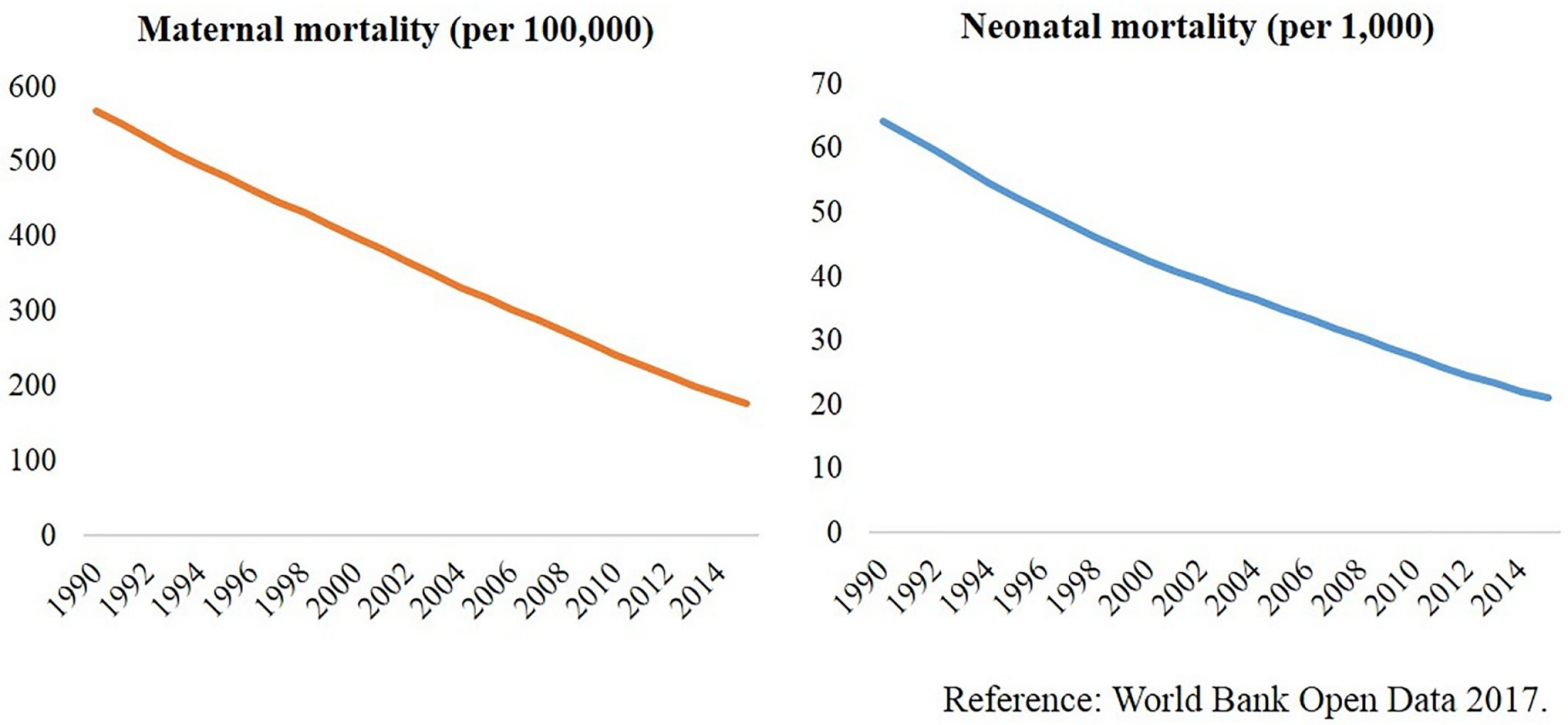
Significant achievement in reduction of maternal and neonatal deaths.

Bangladesh has achieved a significant progress on reduction of maternal and child mortality in the Millennium Development Goal (MDG) era, and also left issues and lessons in the era of post-2015 (Fig 1) [1,2]. The government of Bangladesh has taken maternal and neonatal health and underlying accessibility and quality of healthcare into account, with the global targets of the Sustainable Development Goals (SDGs) to reduce the maternal mortality ratio to less than 70 per 100,000 live births and to reduce the neonatal mortality rate to as low as 12 per 1,000 live births by 2030 [3,4].

Although utilization of essential maternal healthcare services (MHS) including family planning, antenatal care (ANC), postnatal care (PNC), facility-based delivery or delivery by skilled birth attendant (SBA), and emergency obstetric care (EmOC) are proved to reduce maternal mortality [5–7], it has been remarkably reported in many studies and official reports that Bangladesh needs to promote skilled attendance at birth as well as a continuum of care for mothers and babies [1, 8–10]. Underlying this essential issue, the greatest challenge lies in a shortage of human resource, deficiency and poor performance of public healthcare facilities, and mobilization of community particularly in rural areas [1]. The efforts of providing quality services from public healthcare facilities are often offset by a shortage of human resources, inadequate drug supplies and logistics, and weak management of health facilities [11]. As for the demand side, besides wealth status of the households, socio-cultural determinants such as low socio-economic status and resulting social marginalization of women and lack of decision-making power over their lives prevent many women from seeking MHS [12]. Reasons for under-use of existing public health facilities are complex, while it is important to remove physical barriers to access, improve the quality of MHS, and address negative perceptions of rural residents on the government service providers [9, 13–15]. Due to limitations in economic conditions, transportation and social infrastructure, a substantial proportion of delivery still occur at home [16,17], suggesting that gaining understanding and support from community to promote safe childbirth is of high priority. Previous studies strongly proved the effectiveness of community-based interventions to improve maternal and neonatal health outcomes in Bangladesh and other similar settings [7,18]. Besides strengthening of healthcare services through the community-based approach, reduction of maternal and neonatal mortality should be aided by a comprehensive development plan in the rural areas, including education and poverty reduction [9].

Community based interventions, in particular, empowering women’s groups can potentially be cost-effective approaches to prevent maternal and neonatal deaths in the developing countries. The meta-analysis to evaluate the effect of women’s group in reducing maternal and neonatal mortality concluded that women’s group with the application of participatory learning and action (PLA) can contribute to change the women’s behavior during delivery and post-delivery, especially those who participated in the group activities [19]. In those studies in India, Malawi, Nepal, and Bangladesh, women’s groups were formed, the group members participated in educational meetings, and strategies and activities to improve maternal and neonatal health were developed and implemented by themselves. However, those interventions designed with women’s groups were often tried in the research controlled settings, which tend to rely on external resources for implementation, thereby raising a question of scalability and sustainability [20]. The study of Tripathy et al. found statistically significant reduction of neonatal mortality in the intervention clusters with the participatory women’s group [20]. The uniqueness of this study was that the women’s group activities were facilitated by the government community health workers. This study demonstrated that the intervention of women’s group meetings following the PLA cycle can work to prevent neonatal deaths even within the framework of government health systems.

### The community clinic system in Bangladesh

Community clinics (CCs) were introduced by the Government of Bangladesh in 1998 to provide primary health care (PHC) services at the door steps of rural communities, with the service coverage of around 6,000 populations per CC. Since then, the number of CCs reached more than 13,000 nationwide including remote areas but excluding the urban areas. The CC initiative was designed to stimulate community engagement in healthcare: its constructed land was donated by a community beneficiary, and daily operation is managed by a community group. Thus, the CC program is regarded as a model of public-private partnership in the health sector of Bangladesh. A Community Health Care Provider (CHCP) along with other two field staff (Health Assistant and Family Welfare Assistant) are responsible for provision of PHC services at CCs, particularly essential maternal (there are CCs offering normal delivery services without complications), newborn, and child health, and family planning services.

A Community group (CG) serves as the governing body to make sure organization, management, maintenance, and the quality of care at the CC. Three Community Support Groups (CSGs) are established under each CC for demand creation and mobilizing the community in collaboration with a CG. The detailed guidelines of the CG and CSG have been developed by the government, which set the tasks and membership of CG and CSG. Both groups consist of 15–17 community members who represent different social categories such as housewives, persons with disabilities, elderlies, adolescents, religious leaders, and freedom fighters who fought for the country’s independence.

### Safe Motherhood Promotion Project (SMPP)

Aiming to improve maternal and neonatal health outcomes, the Government of Bangladesh has committed to implement strategies targeting to enhance human resource capacities, expand comprehensive EmOC facilities in more sub-district level health facilities, and mobilize communities to stimulate demand and awareness with active involvement of various donors and stakeholders [1,4]. Safe Motherhood Promotion Project (SMPP) jointly implemented by the Government of Bangladesh and Japan International Cooperation Agency (JICA) was a good example to improve the health status of pregnant and postpartum women and neonates through health system strengthening in the rural area [21–25]. A package of interventions named “Narsingdi Model” was implemented following three key strategies: advocacy at the central level, strengthening of public healthcare facilities, and empowerment of community. At the community, a community support system was established to enable community people to organize themselves and implement the activities to promote safe motherhood. Participatory development of an action plan to improve health service delivery and healthy behavior, capacity building of community based groups, case monitoring and management, strengthening of the referral system, improvement of union level health facilities, promotion of health education and collaboration with local government bodies were implemented. As indicated by the impact analysis of SMPP Phase I, the community-based activities promoted women’s access to and knowledge of maternal health care [22]. Taking the SMPP experiences under consideration, the Community Clinic program of the Government of Bangladesh ordered in 2010 to institutionalize the CSGs for increasing the utilization of CC services.

On successful completion of SMPP Phase I, the Bangladesh Government expanded the SMPP as SMPP Phase II in three districts including Satkhira district during July 2011 to June 2016. The objective of SMPP Phase II was to improve maternal and neonatal health outcomes by: improving maternal and neonatal health (MNH) service delivery at health facilities; strengthening CSGs to implement community led actions for saving mother and newborn lives; involving the local government bodies to support MNH services; and empowering women, through awareness building, to obtain participation and accountability for overcoming barriers to access healthcare services. CARE Bangladesh supported JICA to implement the community level activities for both SMPP Phase I and II.

By a cluster randomized controlled trials (RCT), the solid design to prove effectiveness of interventions, the objective of the study was to evaluate the impact of CSG intervention on utilization of maternal and neonatal care services provided by skilled providers and healthcare facilities. Furthermore, this study tried to explore if the CSG, a different type of community based organization from women’s group, can be effective in improving the uptake of MNH services in the rural community.

## Methods

### Study sites and study design

**Fig 2:**
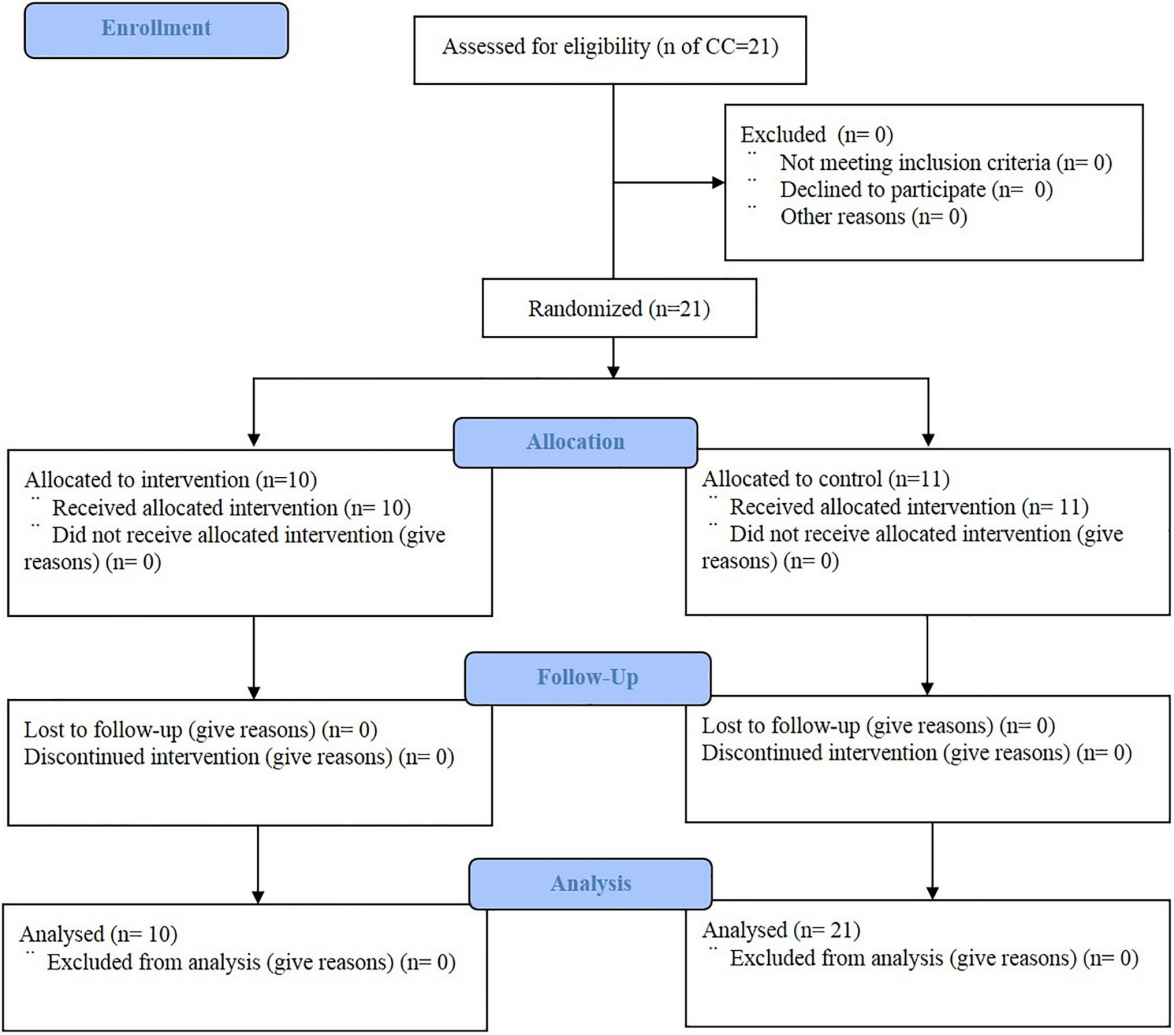
CONSORT flow diagram.

A cluster RCT study was designed to evaluate the impact of CSG intervention of SMPP Phase II in Kalaroa, an upazila (sub-district) of Satkhira District located in the south-west part of Bangladesh (Fig 2). Satkhira District lies with the West Bengal of Indian border and the Bay of Bengal with the population of 2,079,884 according to the census of 2013. Agriculture and fishery are popular sources of earning among local residents.

The baseline survey was conducted in 2012 and the end-line survey was conducted in 2014–15 on completion of the piloting period. The two surveys collected data from 2,339 and 2,336 women who reside in Kalaroa upazila and gave birth in the preceding year of data collection (between 1 June 2011 and 31 May 2012 and between 1 July 2014 and 30 June 2015, respectively). The unit of randomization in this study was a CC in unions (the lowest administrative unit of local government) that covers about 6,000 population in its catchment area.

For the baseline and end-line surveys, a structured pre-coded questionnaire was used for the face-to-face interview. Before the interview we obtained informed written consent from all study participants, in case of the minors also from the husband or guardian resp. Demographic and household data were obtained on all births during the previous 12 months. Information on care seeking behaviors related to antenatal, delivery and postpartum service utilization and newborn practices were obtained to get baseline and end-line estimates.

This study was approved by the IRB of State University of Bangladesh and National Center for Child Health and Development, Tokyo, Japan.

### Participants / Study population

The intervention and comparison areas covered a total population of 147,665 with an estimated birth of 2,657 every year. The study participant eligibility criteria was: women who had a birth during one year preceding the baseline and end-line data collection, resided in the intervention and control areas, and agreed to participate in the study with the written informed consent. Table 1 shows the details of the unions, CCs, and population.

**Table 1:** Details of study site.

Interventional area				Comparison area			
Union	Community clinic	Population	Estimated annual birth	Union	Community clinic	Population	Estimated annual birth
**Sonabaria**	Ram Krishnapur	6,273	125	**Karagachi**	Hatatgonj	7,502	150
**Jugikali**	Bamankali	5,892	118	**Joynagar**	Dhandia	5,630	113
**Chandanpur**	Boyerdanga	8,781	176	**Halatola**	Domdom	8,160	163
**Dayara**	Dayara	8,456	169	**Jugikali**	Ofapur	5,086	102
**Dayara**	Chotosolimpur	7,516	150	**Jalalabad**	Jalalabad	5,358	107
**Halatola**	Halatola	8,350	167	**Sonabaria**	Vadiali	6,730	135
**Karalkata**	Singa	6,000	120	**Karalkata**	Nakila	8,879	178
**Jalalabad**	Batra	6,971	139	**Kusudanga**	Mohammadpur	6,810	136
**Kusudanga**	Paniquria	5,681	114	**Langolzara**	Talkupi	6,540	131
**Karagachi**	Kakdanga	7,223	144	**Chandanpur**	Kadpur	8,235	165
				**Chandanpur**	Hijaldi	9,327	187
**Total**		**71,143**	**1423**	**Total**		**76,522**	**1565**

The sample size was determined to observe mean difference in ANC for at least 4 times between the two groups with an assumption of 20% increase from the baseline and the control with two sided type I error of 0.05 and 80% power. Intra-cluster correlation was assumed to be 0.015. The annual crude birth rate in the population was estimated conservatively (because Satkhira district is a better performing district for use of contraception) according to the Bangladesh Demographic Health Survey 2011, approximately 20 per 1,000 population [26]. As the estimated sample size was close to the total number of the expected births, we included all eligible women in the areas. Consequently, data were collected from a total of 4,675 women, of which 2,339 (intervention: 1,102; comparison: 1,237) in the baseline and 2,336 (intervention: 1,305; comparison: 1,031) in the end-line survey (Table 2).

**Table 2:** Socio-demographic characteristics of the participants in the intervention and control groups.

		Total	Intervention	%	Control	%	*p*
**Number of participants**		4,675	2,407		2,268		
	**Baseline**	2,339	1,102	45.78	1,237	54.54	
	**Endline**	2,336	1,305	54.22	1,031	45.46	
**Age**							0.091
	<15 years	27	11	0.46	16	0.71	
	15–24 years	2,740	1,376	58.61	1,364	60.14	
	25–34 years	1,719	924	38.39	795	35.05	
	>=35 years	189	96	3.99	93	4.10	
**Maternal education**							0.204
	<6 years	985	501	20.81	484	21.34	
	6–8 years	1,807	923	38.35	884	38.98	
	9–11 years	1,313	685	28.46	628	27.69	
	>=12 years	392	298	12.38	272	11.99	
**Marriage**							0.535
	Married	4,639	2,389	99.25	2,250	99.21	
	Separated	24	14	0.58	10	0.44	
	Divorced	7	2	0.08	5	0.22	
	Widowed	5	2	0.08	3	0.13	
**Religion**							0.553
	Islam	4,453	2,289	95.14	2,164	95.41	
	Hinduism	197	102	4.24	95	4.21	
	Christianity	24	15	0.62	9	0.51	
**Occupation**							0.88
	Housewife	4,469	2,302	95.64	2,167	95.55	
	Others	206	105	4.36	101	4.45	
**Education of husband**							0.227
	<6 years	1,599	777	32.28	822	36.24	
	6–8 years	875	448	18.61	427	18.83	
	9–11 years	855	458	19.03	397	17.50	
	>=12 years	1,346	724	30.08	622	27.43	
**Occupation of husband**							<0.001
	Business	887	493	20.56	394	17.53	
	7.Agriculture	1,275	594	24.77	681	30.29	
	Skilled labor	574	281	11.72	293	13.03	
	Unskilled labor	690	363	15.14	327	14.55	
	Rickshaw/van puller	306	157	6.55	149	6.63	
	Others	914	510	21.27	404	17.97	
**Household income (month)**							0.219
	1st quintile (<=5000 Taka)	1,227	636	26.44	591	26.07	
	2nd quintile (5000–6000 Taka)	770	383	15.93	387	17.07	
	3rd quintile (6000–8000 Taka)	1,066	529	22.00	537	23.69	
	4th quintile (8000–10000 Taka)	738	382	15.88	356	15.70	
	5th quintile (>=10000 Taka)	871	475	19.75	396	17.47	
**Number of living children**							0.198
	0	37	19	0.79	18	0.79	
	1	2,262	1,138	47.28	1,124	49.56	
	2	1,664	892	37.06	772	34.04	
	>=3	712	358	14.87	354	15.61	

### Randomization and masking

Kalaroa upazila (sub-district) consists of 12 unions, and there are 21 CCs in 11 unions (one union is excluded due to its urban location). All the 21 CCs were randomized to allocate 10 CCs for intervention and 11 CCs for comparison, as showed in Table 1. In the intervention area, the intervention as described below was implemented, while in the control area, the existing government service package continued. Due to the natural characteristic of the intervention, the participants could not be masked to their study group allocation, while the trained data collectors were blinded about the objectives of the study.

### Interventions

The intervention was designed as a part of the SMPP Phase II implemented in Satkhira District, which included activities at government healthcare facilities and community levels and bridging the community with health facilities. The main activities were: i. Community diagnosis and resource mapping exercise, ii. Advocacy and planning meetings at union level, iii. Establishment of CSGs, iv. Capacity building of CSG members and Union Parishad (local government) for implementing community mobilization activities, v. Promoting birth planning, ANC, PNC, and neonatal care counseling and timely referral through engaging selective female CSG members and mobilizing local resources, vi. Maternal and perinatal death audit at the community level, and vii. Enhancement of maternal and neonatal health-related knowledge and practices among the residents. The community level intervention was primarily facilitated by CARE Bangladesh.

In the intervention areas, CSGs were developed under the leadership of CG members who received training and facilitation supports from the project staff. The key functions of the CSG members included: i. Registration of pregnant women, tracking and follow up of continuum of care for mother and babies; ii. Promotion of birth planning, ANC, PNC and safe delivery; iii. Identification and referral of complications of pregnant and postpartum women and newborns; iv. Mobilization of local resources to support pregnant and postpartum women and newborns for referral and use of life saving services; and v. Development of mechanism for coordination, linkage and sharing information with CC, Family Welfare Center, and Upazila Health Complex (UHC).

The SMPP Phase II also supported development of selective female members of CSGs as volunteers (unpaid) by providing training and on the job assistance through regular monitoring. Female CSG volunteers were trained on essential MNH care and worked for registration of pregnant women through household visits in their assigned areas. During household visits, the volunteers organized birth planning sessions with pregnant women and their family and encouraged pregnant women to take ANC service and use skilled assistance for childbirth. The volunteers also followed up the women after the delivery and refer them to a skilled provider or health facility, if necessary. The volunteer activities were shared and discussed at the CSG bi-monthly meeting.

Based on the operational guideline of the government, CSGs were established and strengthened in the intervention areas. On the other hand, in the comparison areas, the CGs were trained only on management of CCs but not on development of CSGs. It was observed that CSGs were formed but not functional in the comparison areas.

### Outcomes

The expected outcome of this study was increased utilization of services for antenatal, delivery, postpartum and neonatal care by the pregnant and post-partum women. We examined the proportion of women received those services provided by skilled health personnels including Community-based Skilled Birth Attendants (CSBA), Family Welfare Visitors (FWV), nurses and doctors, and at health facilities (both public and private).

The major indicators of the expected outcomes were:

- Proportion of women received any and 4+ ANC from skilled health care providers;
- Met need (proportion of women with complications received services from EmOC facilities) during pregnancy, childbirth and post-partum period;
- Delivery attended by skilled birth attendants;
- Delivery conducted at health facilities;
- Proportion of postpartum women received PNC from skilled providers within 42 days of delivery;
- Proportion of sick newborns received services from skilled provider

Information related to maternal and neonatal complications and care seeking were obtained from the respondents through face-to-face interview using a structured questionnaire.

### Statistical analysis

Data of baseline and end-line surveys were combined and analyzed using Stata 14.1. Analyses were based on the intention-to-treat principle and compared differences of the outcomes between the intervention and control at both individual and cluster levels. First, univariate analyses were performed to explore the characteristics of respondents. In the comparison of each variable, a stratification by cluster was implemented to examine the equality of covariates of the two groups at baseline. To identify the differences in the two groups over time, we performed a difference-in-difference analysis (DID) with adjustment for covariates including maternal age, education, occupation, wealth status presented by monthly income quintiles, number of living children, and CCs (cluster). The DID estimator, a coefficient of the change between intervention (0 for control and 1 for intervention) and time (0 for baseline and 1 for end-line), showed the impact of intervention. To adjust correlations of the outcome indicators in the cluster, generalized mixed effects regression models were employed to estimate the relative risk (RR) as a measure of intervention effect and to adjust the effects of baseline variables at the individual level [27, 28]. The major outcome variables measured at the different times are adjusted for the baseline value, and to assess the effect of the intervention, time (baseline and end-line), intervention and interaction between the intervention and the time are added to the model. The equation is described below [28]:

$${\text{Y}}_{\text{t}}=\beta_{0}+\beta_{1}\text{X}+\beta_{2}{\text{Y}}_{\text{t}0}+\beta_{3}\;\text{time}+\beta_{4}\text{X}\times \text{time}$$

Where the regression coefficient for the intervention variable reflects the intervention effect at the end-line, and β_4_ is the coefficient for the interaction between the intervention and the time.

For the equity of utilization, we also examined the RR by wealth quintiles. Appendix showed an example of stata script. All results of the statistical analysis were presented with 95% confidence interval (CI).

The RCT was registered at the UMIN Clinical Trial Registry (UMIN000031789). The project started without any specific academic interest. When we recognized the achievements of our intervention we decided to seek publication and therefore registered the trial (retrospectively).

CONSORT checklist is presented as Supplementary Document.

### Role of the funding source

The funding source did not affect the conduct, analyses or results of the study.

## Results

A total of 2,407 (1102 in baseline and 1305 in end-line) and 2,268 (1237 in baseline and 1031 in end-line) women were enrolled in the intervention group and control group, respectively, meeting the requirement of sample size as planned. Table 2 showed the socio-demographic characteristics of the participants in the two groups. Except husband’s occupation, the distribution of socio-demographic characteristics did not differ significantly between the two groups. On an average, the participants were aged 23.7 years (SD: 4.98 years), with 7.68 years of education (SD: 2.62 years) and 1.69 children (SD: 0.86 children). The enrollment rate did not vary across unions and times.

The average frequency of the women received ANC is 3.73 times (SD: 2.13 times), 4.43 times (SD: 1.89 times), 4.04 times (SD: 2.19 times) and 4.57 times (SD: 2.06 times) in the baseline of the intervention, the end-line of the intervention, the baseline of the control, and the end-line of the control, respectively.

**Fig 3:**
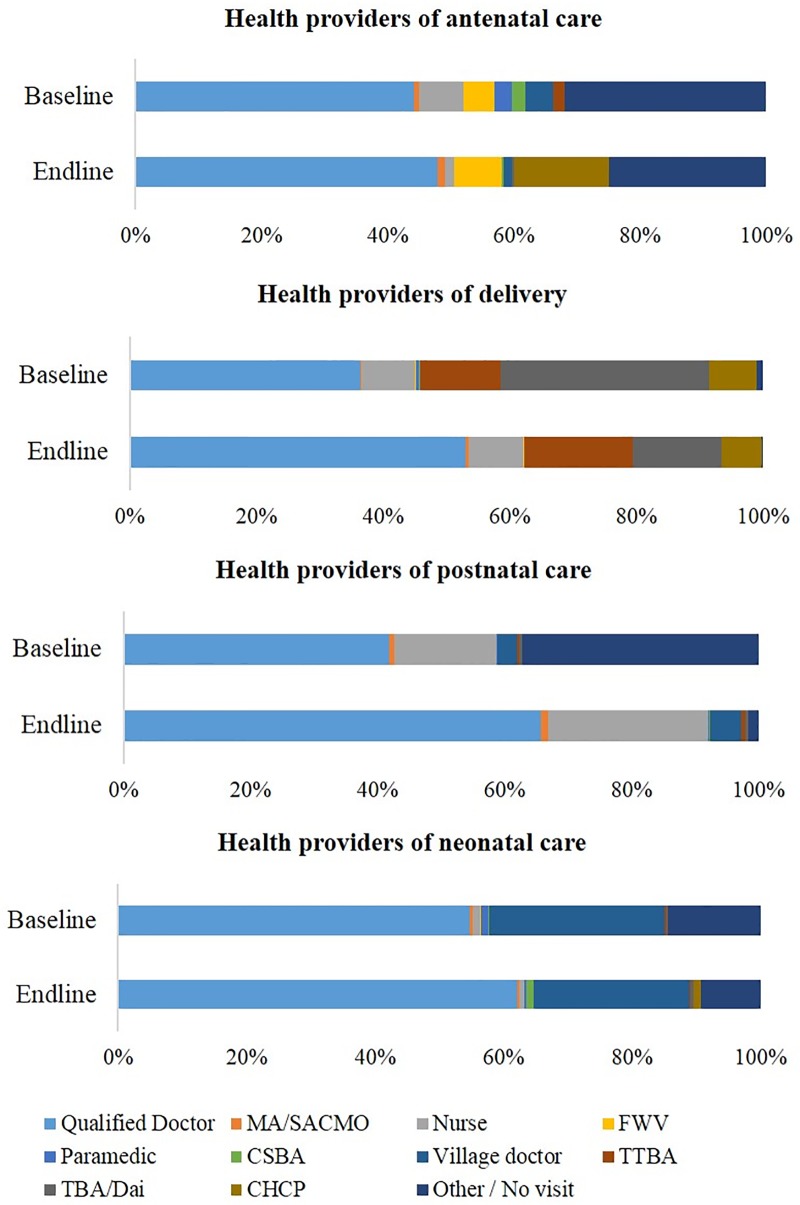
Health providers for maternal and neonatal care at baseline and end-line.

**Fig 4:**
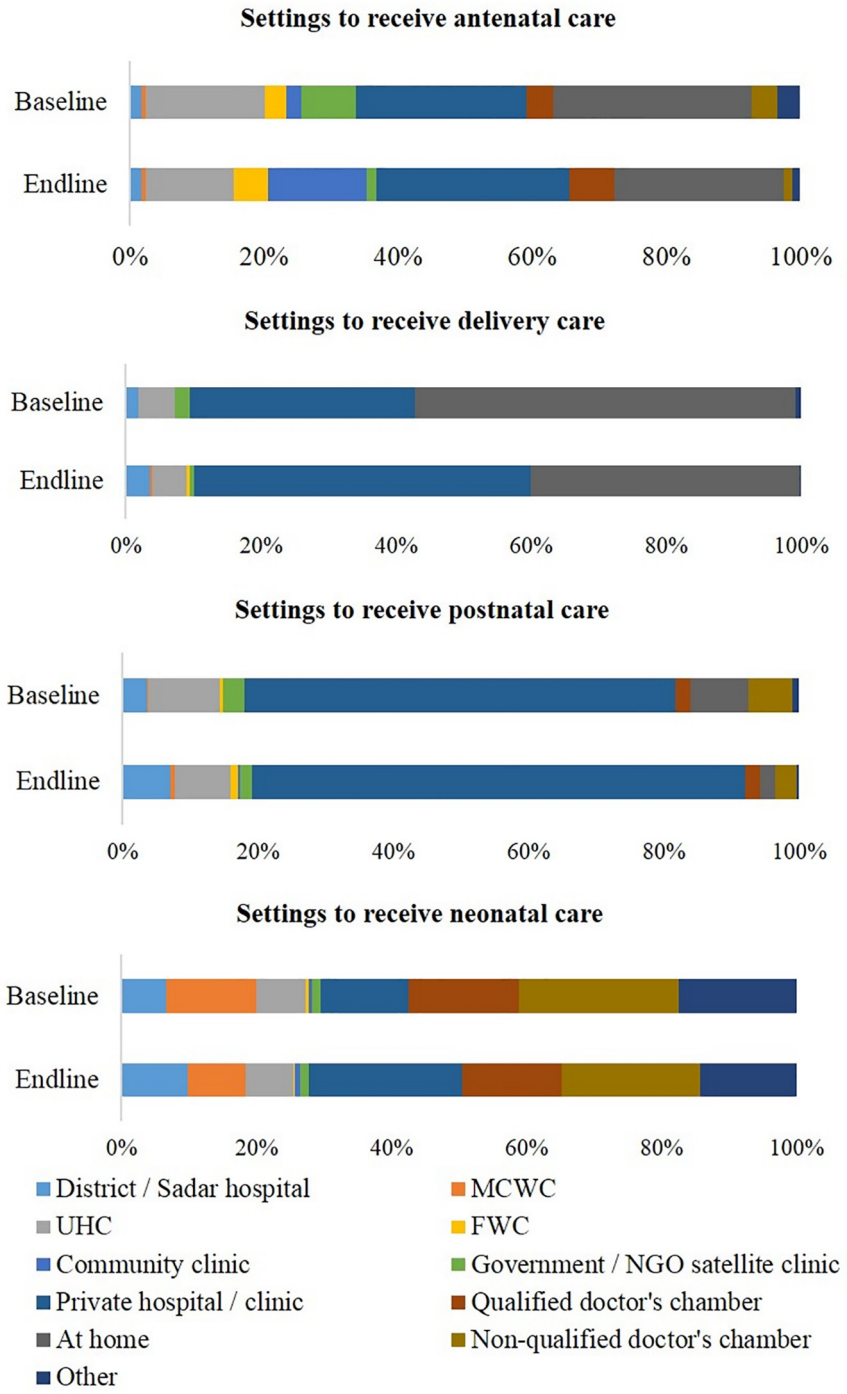
Settings for maternal and neonatal care at baseline and end-line.

Figs 3 and 4 summarized the details of maternal and neonatal care providers and facilities / settings at the baseline and end-line of intervention. Qualified doctors and private health facilities served as the major provider for ANC, delivery care, PNC and neonatal care. Regarding the place of birth, delivery at private health facilities increased from 33.2% at the baseline to 49.9% at the end-line in the intervention areas. Home delivery decreased largely, with the proportion of 39.8% at the end-line of the intervention compared to that of 56.4% at the baseline. The visiting of CCs for ANC increased from 3.3% to 25.4%. As for sick newborn care, 38.4% women did receive services from facilities or skilled health care providers.

Multilevel DID analysis shows significant improvements in utilization of ANC provided by skilled health personnel, delivery attended by skilled healthcare providers and facility-based delivery, utilization of emergency obstetric care (EmOC) services for antenatal and postnatal complications, and PNC by skilled provider during 42 days of delivery (Table 3). Although there was no significant difference in the incidence of complications during pregnancy and delivery, an increase of proportion of cesarean section was observed after the intervention (DID estimator = 0.076).

**Table 3:** Difference-in-Difference analysis of the utilization of maternal and neonatal care.

		Intervention (%)		Control (%)		DID, unadjusted	DID, adjusted
		Baseline	Endline	Baseline	Endline		
**Skilled ANC**	No	211 (19.15)	173 (13.26)	125 (10.11)	218 (21.14)		
	Yes	891 (80.85)	1132 (86.74)	1,112 (89.89)	813 (78.86)	0.169 ***	0.165 **
**Skilled ANC 4**	No	529 (48.00)	417 (31.95)	526 (42.52)	314 (30.46)		
	Yes	573 (52.00)	888 (68.05)	711 (57.48)	717 (69.54)	0.040	0.038
**Skilled ANC 6**	No	823 (74.68)	916 (70.19)	865 (69.93)	704 (68.28)		
	Yes	279 (25.32)	389 (29.81)	372 (30.07)	327 (31.72)	0.028	0.026
**Facility complication in pregnancy**	No	147 (29.82)	54 (15.98)	134 (22.34)	57 (21.43)		
	Yes	346 (70.18)	284 (84.02)	449 (77.02)	209 (78.57)	0.123 **	0.120 **
**Skilled delivery**	No	599 (54.36)	490 (37.55)	613 (49.56)	450 (43.65)		
	Yes	503 (45.64)	815 (62.45)	624 (50.44)	581 (56.35)	0.109 ***	0.101 **
**Facility delivery**	No	631 (57.26)	522 (40.00)	677 (54.73)	471 (45.68)		
	Yes	471 (42.74)	783 (60.00)	560 (45.27)	560 (54.32)	0.082 **	0.074 *
**Facility complication in delivery**	No	117 (21.75)	19 (4.03)	125 (20.87)	18 (5.47)		
	Yes	421 (78.25)	452 (95.97)	474 (79.13)	311 (94.53)	0.023	0.024
**Facility complication in postpartum**	No	62 (28.97)	15 (8.57)	62 (22.22)	17 (13.71)		
	Yes	152 (71.03)	160 (91.43)	217 (77.78)	107 (86.29)	0.119 *	0.115
**Skilled 42**	No	714 (64.79)	493 (37.78)	715 (57.80)	451 (43.74)		
	Yes	388 (35.21)	812 (62.22	522 (42.20)	580 (56.26)	0.13 ***	0.123
**Skilled baby 28**	No	101 (15.35)	54 (10.27)	115 (15.21)	53 (12.05)		
	Yes	557 (84.65)	472 (89.73)	641 (84.79)	387 (87.95)	0.019	0.018
**Breastfeeding early**	No	790 (71.69)	800 (61.30)	888 (71.79)	628 (60.91)		
	Yes	312 (28.31)	505 (38.70)	349 (28.21)	403 (39.09)	-0.005	-0.003
**Breastfeeding exclusive**	No	918 (83.68)	281 (21.53)	1,029 (83.39)	212 (20.56)		
	Yes	179 (16.32)	1,024 (78.47)	205 (16.61)	819 (79.44)	-0.007	-0.008
**Mode of delivery**	Normal	727 (65.97)	642 (49.2)	771 (62.33)	568 (55.09)		
	Cesarean Section	353 (32.02)	646 (49.5)	420 (33.95)	452 (43.84)		
	Others	22 (1.99)	17 (1.3)	46 (3.72)	11 (1.07)	0.084 **	0.076 *
**Complications during pregnancy**	No	609 (55.26)	967 (74.10)	654 (52.87)	765 (74.20)		
	Yes	493 (44.74)	338 (25.90)	583 (47.13)	266 (25.80)	0.023	0.024
**Complications in delivery**	No	564 (51.18)	833 (63.83)	638 (51.58)	700 (67.90)		
	Yes	538 (48.82)	472 (36.17)	599 (48.42)	331 (32.10)	0.037	0.034
**Complications in postnatal period**	No	888 (80.58)	1,130 (86.59)	958 (77.45)	907 (87.97)		
	Yes	214 (19.42)	175 (13.41)	279 (22.55)	124 (12.03)	0.045 *	0.046
**Complication of baby in neonatal period**	No	444 (40.29)	779 (59.69)	481 (38.88)	591 (57.32)		
	Yes	658 (59.71)	526 (40.31)	756 (61.12)	440 (42.68)	-0.010	-0.006
**Utilization of CC for ANC**	No	982 (96.75)	951 (74.59)	1073 (91.79)	754 (74.88)		
	Yes	33 (3.25)	324 (25.41)	96 (8.21)	253 (25.12)	0.052*	0.055

*p<0.05,

**p<0.01,

***p<0.001

Table 4 summarized the relative risk for utilization of maternal and neonatal care services to show the effects of intervention, after adjustment for covariates of individuals and clusters. As the wealth status, presented by monthly income quintiles, significantly modified the outcomes in logistic regression models at the individual level and in generalized mixed effects regression models, analysis of those indicators of healthcare seeking behaviors was stratified by income quintile. The effects of intervention were especially observed in the 2^nd^ and 3^rd^ income quintile (5000 to 6000 and 6000 to 8000 Taka per month, respectively), as healthcare seeking in more items, including ANC provided by skilled healthcare providers, delivery attended by skilled healthcare providers and facility-based delivery, utilization of EmOC for antenatal and postnatal complications, and PNC provided by skilled providers during 42 days of delivery, showed a significant improvement after the intervention.

**Table 4:** Adjusted relative risk for utilization of maternal and neonatal care services by wealth quintiles.

	Overall	1st quintile	2nd quintile	3rd quintile	4th quintile	5th quintile
**Skilled ANC**	3.74 (2.67–5.22)***	2.29 (1.24–4.23)**	8.39 (3.78–18.60)***	3.69 (1.84–7.42)***	3.41 (1.28–9.07)*	19.60 (5.10–75.32)***
**Skilled ANC 4**	1.14 (0.89–1.46)	0.83 (0.50–1.38)	0.87 (0.48–1.61)	1.87 (1.10–3.18)*	1.52 (0.80–2.89)	1.16 (0.63–2.13)
**Skilled ANC 6**	1.10 (0.85–1.44)	0.85 (0.49–1.46)	0.81 (0.41–1.58)	1.61 (0.89–2.93)	1.42 (0.73–2.75)	1.31 (0.70–2.45)
**Facility complication in pregnancy**	1.90 (1.12–3.20) *	0.39 (0.12–1.26)	6.08 (1.90–19.47)**	3.43 (1.17–10.03)*	3.44 (0.91–13.08)	1.92 (0.51–7.22)
**Skilled delivery**	1.52 (1.19–1.94) **	0.95 (0.58–1.55)	2.90 (1.58–5.32)**	2.09 (1.26–3.48)**	1.22 (0.68–2.24)	1.55 (0.82–2.94)
**Facility delivery**	1.35 (1.06–1.72) *	0.86 (0.53–1.41)	2.58 (1.41–4.71)**	1.90 (1.15–3.15)*	1.11 (0.61–2.04)	1.12 (0.61–2.09)
**Facility complication in delivery**	1.69 (0.80–3.57)	1.58 (0.26–9.50)	7.29 (1.13–47.11)*	3.04 (0.65–14.26)	0.41 (0.04–4.42)	0.84 (0.14–4.95)
**Facility complication in postpartum**	2.61 (1.07–6.36) *	5.45 (0.43–68.80)	3.52 (0.26–47.67)	0.85 (0.12–5.90)	3.32 (0.49–22.41)	2.82 (0.19–42.19)
**Skilled 42**	1.61 (1.25–2.06) ***	0.92 (0.55–1.55)	2.70 (1.45–5.05)**	3.07 (1.81–5.21)***	1.51 (0.82–2.81)	1.20 (0.64–2.24)
**Skilled baby 28**	1.18 (0.71–1.96)	1.32 (0.51–3.42)	0.68 (0.21–2.19)	1.10 (0.36–3.38)	1.65 (0.37–7.33)	2.15 (0.58–7.97)
**Breastfeeding early**	1.03 (0.80–1.32)	1.93 (1.17–3.21)*	0.50 (0.27–1.94)	0.66 (0.39–1.14)	1.53 (0.81–2.90)	0.91 (0.48–1.73)
**Breastfeeding exclusive**	0.93 (0.68–1.26)	0.81 (0.43–1.53)	2.71 (1.27–5.76)*	0.80 (0.43–1.51)	0.42 (0.19–0.95)*	0.88 (0.42–1.86)
**Mode of delivery**	1.39 (1.09–1.78)**	0.90 (0.54–1.48)	2.22 (1.19–4.14)*	2.10 (1.25–3.54)**	1.20 (0.64–2.25)	1.36 (0.76–2.46)

*p<0.05,

**p<0.01,

***p<0.001

## Discussion

To our knowledge, this is the first cluster randomized controlled trial to evaluate the CSG intervention of SMPP in rural Bangladesh. Our findings showed that the intervention through development and strengthening of CSGs and community mobilization significantly improved the utilization of antenatal, delivery and postnatal care services, after adjustment of covariates at the individual and cluster levels. In the project, the CSG members built a capacity to coordinate participatory approaches to implement the group activities and persuaded pregnant women for accessing skilled MHC services. CSGs share some similarities with women’s groups, of which effectiveness in reducing neonatal and maternal mortalities has already been proven by several studies as discussed earlier [19]. For example, the functions of CSG such as organizing meetings and developing action plans are grounded in a participatory approach with a critical role played by facilitators. On the other hand, CSGs differ from women’s groups: one of such differences is that a CSG is a key component of the government endorsed and supported community mobilization mechanism to promote the utilization of CCs. As per the government policy, CSGs have already been expanded throughout the country. In this context, our study contributed for the government, by evaluating the effect of CSGs in improving maternal and neonatal health, to determine the future direction of the government run Community Clinic program, specifically on how to engage community people for their own well-beings. Another difference is active participation of male CSG members in the discussions and activities related to maternal and neonatal health. We observed comparative advantages of male members over female counterparts in terms of approaching men for behavior change and mobilizing community resources including fund raising and emergency supports. Nevertheless, the male membership in the group could be challenged by negative opinions: women’s groups can openly discuss women’s issues and concerns with the absence of men in the group while female members of CSGs may feel shy to share their personal feelings and experiences in front of male members. We did not evaluate under this study; yet, the question of influence of male participation in the group activities could be a potential area for future studies.

Though our study purposefully focused on the impact of CSG intervention to be evaluated, the overall SMPP Phase II interventions, which the intervention of this study was a part of, were comprised of interacting interventions to facilitate safe motherhood practices covering the women’s reproductive cycle including antenatal, delivery and postnatal period and their babies, and integrated the two approaches to address the demand and to improve the supply of MHC services at the community level and its referral facilities [29]. Building on the lessons learned from the SMPP phase I along with the findings of previous studies, an integrated community-based strategy combined with health system strengthening is likely to be an effective intervention to improve the maternal and neonatal health in the developing countries [7, 19, 30, 31].

Additionally, an equity perspective in policy interventions is crucial to realize safe motherhood and childbirth [32]. In our study, the impacts of the intervention were in particular significant among the women of 2^nd^ and 3^rd^ quintiles of household wealth status, proving that the integrated community-based strategy with health system strengthening is also effective to achieve equity in particular for the low- and middle-income households [33].

With improvement of knowledge and awareness on MHC by the intervention, our results identified changing health seeking behaviors during pregnancy and childbirth. After the intervention, the utilization of qualified healthcare facilities increased, especially that of CCs for ANC and private hospitals / clinics for all maternal and neonatal care. Although approximately only a quarter visited CCs for ANC is still low, the largely increased figure compared to the baseline proved a potential to change local people’s awareness and perception on public health services by the integrated community strengthening approach, as previously suggested [34]. Improving the perception and satisfaction on skills and service providers’ attitude towards patients, quality of care, logistics supply, and physical environment is crucial for the utilization of CCs and other public health facilities as well, besides socioeconomic status such as education and income [35].

It is likely to explain the result that although the utilization of district hospital slightly increased after the intervention, private hospitals / clinics have become the major provider of maternal and neonatal care services [36]. Compared to the public counterpart, private hospitals / clinics were more likely to be equipped for EmOC [37–40]. On the other hand, the government-driven public services are more affordable, accessible for the location, and ensure the equitable utilization in the community, particularly for the poor [37,40]. No evidence in either Bangladesh or elsewhere of low- and middle-income countries (LMICs) supported the private sector to be more efficient, accountable, or medically effective [41]. Besides strengthening the primary health system in terms of quantity, incentive and capacity of manpower, environment / infrastructure, quality of care, and building public-private partnership is expected to provide better continuum of care for mothers and babies [38,39]. It is necessary for the resource limited countries including Bangladesh to engage diverse stakeholders in maternal and neonatal health, including private practitioners, local business, NGOs and local government bodies to fill up the resource gaps that undermine health service delivery in the public sector.

Our results suggested the trend of increasing cesarean section (CS) after the intervention. For the rising tendency of the use of CS in Bangladesh in the past decade [42], the reasons are complex; whereas based on our findings and other previous surveys in South Asia, probably attributable to the issue of improved awareness and wealth status, women are more likely to select CS at private hospitals for perceived safety and quality [36,43]. In LMICs, CSs are more likely to be performed for financial benefits, contributing to the unreasonably high rate [44]. This emerging issue urgently needs to be considered in the community-based interventions for safe motherhood based on solid research evidences, to address the factors at both the supply and the demand side.

The main strength of this study is the design of cluster RCT provided strong evidence on the effects of the project. However, we also recognize limitations. First, we did not measure the downstream outcomes of maternal and neonatal mortalities and morbidities as the specific aim of the intervention was to improve the utilization at first. For this, the impacts of the project on population health could not be directly indicated. Moreover, besides the activities at the primary level as showed in this study, the SMPP implemented activities to improve quality of care provided by the health facilities in the entire Satkhira District, potentially causing better results of the major indicators at both the intervention and control groups compared to other studies [45,46]. However, as those measures were at the secondary level, rather than at the community, its influence on the individual and the cluster of the both groups can be considered similar.

## Conclusion

The integrated community-based strategy combined with health system strengthening as foundation effectively improved the utilization of MHS in rural Bangladesh, with perspectives and outcomes in equity. The success suggested a potential of the government efforts to strengthen community support system for ensuring safe motherhood. To provide better maternal and neonatal care, continuous efforts to promote the demand and to address perception and awareness among local people by engaging communities, together with strengthening the public healthcare and building an innovative public-private partnership are necessary. The intervention helped to identify and remove existing and emerging barriers that lie between women and healthcare providers for safe motherhood and a continuum of care.

## Supporting information

S1 Table (PDF)

S1 File (DTA)

S2 File (PDF)

S3 File (TXT)

S4 File (PDF)

S5 File (PDF)
