# Supplementary material for: Strengthening the community support group to improve maternal and neonatal health seeking behaviors: A cluster-randomized controlled trial in Satkhira District, Bangladesh
Source: PLoS One. 2019 Feb 28;14(2):e0212847. doi: 10.1371/journal.pone.0212847 (PMC6394907; doi:10.1371/journal.pone.0212847)
Supplement: S2 File — (PDF) [file pone.0212847.s003.pdf]

Abstract for
Ethical Review Committee

Study title:

Impact Evaluation of Community Support Group under the Safe Motherhood Promotion Project in Bangladesh

Purpose of the study:

The aim of this study is to evaluate the effectiveness of community support groups (CSGs) in improving maternal and neonatal health seeking behavior, women's empowerment and social capital of the community.

Answer to the ethical questions:

1. The study population is recently delivered women (women who had a live birth one year prior to the surveys) and general population (both gender) of the community. The study population does not include children or any other risk group whose voluntary informed consent is questionable.

2. This study does not pose any risk (physical, mental, psychological, social or legal) to the study population. Rather, if the study findings are positive, it would have strong policy implication for the country and all the population would benefit out of this intervention.

3. The study does not have any potential risk for the population.

4. This study will collect data through questionnaire interview and economic games. The data (soft and hard copy) will be handled cautiously so that nobody can have access to the data set except the research team members. The data will only be used for the study purpose and no where in the report the respondents' identity will be stated.

5. Informed consent will be taken from all the respondents. Before interview, all the respondents will be read out a consent statement (see the questionnaire for the informed consent statement) stating the study objectives, their rights (such as, respondents may wish not to answer any question and they can withdraw themselves anytime during the interview), duration of data collection etc. Verbal consent will be taken after the statement is read out to the respondents and data collection will only start if consent is given.

   a) We shall avoid signed consent because it is expected that many of the target groups would be illiterate.

   b) For withheld information, there is no provision of penalty for the respondents. The respondents would have the right to withdraw themselves from the interview any time if they want.

   c) The respondents (household interview) will be approached during the leisure time for interview and compensation is not planned for this. However, the respondents, at the end of the interview, will be provided health information if they have misconceptions about good practices. On the other hand, the respondents who would participate in the economic games will be compensated for their wage loss and transportation cost.

6. The interview would take place at the respondents' house when she is free/ or at the time provided by the respondents. They will never be forced to give the interview. The approximate length of time for the interview would be 30 minutes.

7. If this study is found to be positive, it would have impact on reduction of maternal and neonatal morbidity and mortality through improvement of health seeking behavior, utilization of services, women's empowerment and social capital, which the Bangladesh Government is keen to achieve. Moreover, it would also contribute to the global learning on good practices.

8-11: This study is not intended for drug trial. Therefore, these questions are not relevant for this study.

12. No, this study does not require to collect information from the hospital records related to birth, death, etc. and involve any kind of biological samples such as organs, tissue, fetus or the abortus.
