# Supplementary material for: Strengthening the community support group to improve maternal and neonatal health seeking behaviors: A cluster-randomized controlled trial in Satkhira District, Bangladesh
Source: PLoS One. 2019 Feb 28;14(2):e0212847. doi: 10.1371/journal.pone.0212847 (PMC6394907; doi:10.1371/journal.pone.0212847)
Supplement: S4 File — (PDF) [file pone.0212847.s005.pdf]

UMIN-CTR 臨床試験登録情報の閲覧

BACK TOP ● UMIN-CTR ホーム ● 用語の説明(簡易版) ● 用語の説明(詳細版)--準備中 ● FAQ

利用者名： GAI RUOYAN UMIN ID： gai-r

試験進捗状況	参加者募集終了-試験継続中/No longer recruiting
UMIN試験ID	UMIN000031789
受付番号	R000036286
試験名	バングラデシュ国における母性保護サービス強化プロジェクト(SMPP)フェーズ2の評価研究
一般公開日(本登録希望日)	2018/05/31
最終更新日	2019/01/31

※ 本ページ収載の情報は、臨床試験に関する情報公開を目的として、UMINが開設しているUMIN臨床試験登録システムに提供された臨床試験情報です。
※ 特定の医薬品や治療法等については、医療関係者や一般の方に向けて広告することは目的としていません。

基本情報/Basic information		
試験名/Official scientific title of the study	バングラデシュ国における母性保護サービス強化プロジェクト(SMPP)フェーズ2の評価研究	Interventional study on the impact of safe motherhood promotion project Phase II in Bangladesh
試験簡略名/Title of the study (Brief title)	バングラデシュ国におけるSMPP評価研究	JICA SMPP II in Bangladesh
試験実施地域/Region	アジア(日本以外)/Asia(except Japan)	

対象疾患/Condition		
対象疾患名/Condition	母子保健指標	Maternal and neonatal health indicators
疾患区分1/Classification by specialty	該当せず/Not applicable	
疾患区分2/Classification by malignancy	悪性腫瘍以外/Others	
ゲノム情報の取扱い/Genomic information	いいえ/NO	

目的/Objectives		
目的1/Narrative objectives1	バングラデシュ国シャトキラ県コラロア郡で実施されたSMPPの介入の母子保健サービス利用や母子保健指標の改善に関する効果を検証する。	To assess the effectiveness of SMPP intervention in Kalaroa Upazila, Satkhira District on improving maternal and neonatal indicators and service utilization
目的2/Basic objectives2	安全性・有効性/Safety,Efficacy	
目的2 -その他詳細/Basic objectives -Others		
試験の性質1/Trial characteristics_1	検証的/Confirmatory	
試験の性質2/Trial characteristics_2	実務的/Pragmatic	
試験のフェーズ/Developmental phase	該当せず/Not applicable	

評価/Assessment		
主要アウトカム評価項目/Primary outcomes	・研究対象となる妊婦が妊娠期間中に専門の技能を持つスタッフによる健診を4回以上を受ける割合;<br>・専門の技能を持つスタッフの介助による分娩を受ける割合;<br>・施設分娩の割合;<br>・産科救急ケアの利用率;<br>・専門の技能を持つスタッフによる産後ケアの普及率;<br>・新生児の疾患が起こる場合、専門の技能を持つスタッフによる新生児ケアサービスを受ける割合	- Proportion of targeted women (recently delivered women) received 4 or more ANC by skilled providers;<br>- Proportion of deliveries attended by skilled birth attendants<br>- Proportion of deliveries conducted at the health facilities (both public and private)<br>- Proportion of women with complications during pregnancy/delivery/postpartum period utilized EmOC services (met need)<br>- Proportion of targeted women received PNC within 42 days of delivery by skilled providers<br>- Proportion of mother&babies received neonatal care services from trained health providers (or health facilities) during neonatal sickness
副次アウトカム評価項目/Key secondary outcomes		

アウトカム評価項目には、可能な限り評価の時期の情報(例:投与開始から12週後など)も含めてください。また、評価項目としては、単に「安全性」「有効性」などとするのではなく、実際に測定する検査項目の名称を具体的にご記入ください。また、主要アウトカム評価項目は最も主要な1項目のみとし、2項目以降は副次アウトカム評価項目としてください。

基本事項/Base	
試験の種類/Study type	介入/Interventional

試験デザイン/Study design	
基本デザイン/Basic design	並行群間比較/Parallel
ランダム化/Randomization	ランダム化/Randomized
ランダム化の単位/Randomization unit	集団/Cluster
ブラインド化/Blinding	オープンだが測定者がブラインド化されている/Open -but assessor(s) are blinded
コントロール/Control	無治療対照/No treatment
層別化/Stratification	はい/YES
動的割付/Dynamic allocation	いいえ/NO
試験実施施設の考慮/Institution consideration	
ブロック化/Blocking	いいえ/NO
割付コードを知る方法/Concealment	知る必要がない/No need to know

介入/Intervention	
群数/No. of arms	2
介入の目的/Purpose of intervention	教育・カウンセリング・トレーニング/Educational,Counseling,Training

介入の種類/Type of intervention	行動・習慣/Behavior,custom	
介入1/Interventions/Control_1	母子保健サービス提供者のスキル・能力強化;<br>住民の組織化による主体的な安全なお産活動;<br>病院における母子保健サービスの質の改善;<br>地方行政機関との連携による母子保健啓発活動;<br>母子保健関連グッドプラクティスの他地域への普及・政策化	Safe Motherhood Promotion through organizing communities,<br>Improvement of quality MNH services at hospitals,<br>Collaboration with local government bodies to mainstream MNH,<br>Replication and policy development of good practices related to MNH
介入2/Interventions/Control_2	対照群は既存のサービスパッケージを実施	The current service package
介入3/Interventions/Control_3		
介入4/Interventions/Control_4		
介入5/Interventions/Control_5		
介入6/Interventions/Control_6		
介入7/Interventions/Control_7		
介入8/Interventions/Control_8		
介入9/Interventions/Control_9		
介入10/Interventions/Control_10		

介入1～10には、介入の期間（何日間投与、介入するか）、介入の量（投与量や線量など）、介入の回数、頻度など、内容を可能な限り詳しく記載してください。とくに、投薬や機器使用の介入の場合、期間は必ず含めてください。

適格性/Eligibility		
年齢（下限）/Age-lower limit	15 歳/years-old 以上/<=	
年齢（上限）/Age-upper limit	49 歳/years-old 以下/>=	
性別/Gender	女/Female	
選択基準/Key inclusion criteria	対象地域で過去1年に出産を経験した女性（死産を含む）	Women who had child birth during last one year in the targeted areas.
除外基準/Key exclusion criteria	本プロジェクトに参加する意思がなし	Not willing to participate in the project
目標参加者数/Target sample size	2500	

責任研究者/Research contact person		
責任研究者名/Name of lead principal investigator	吉村幸江	Yoshimura Yukie
所属組織/Organization	日本国際協力機構	Japan International Cooperation Agency

所属部署/Division name	バングラデシュ事務所	Bangladesh Office
住所/Address	3rd Floor, Bay's Galleria, 57 Gulshan Avenue, Gulshan 1, Dhaka 1212, Bangladesh	3rd Floor, Bay's Galleria, 57 Gulshan Avenue, Gulshan 1, Dhaka 1212, Bangladesh
電話/TEL	+880-2-989-1897	
Email/Email	yoshiboubd@gmail.com	

試験問い合わせ窓口/Public contact		
試験問い合わせ窓口担当者名/Name of contact person	森臨太郎	Mori Rintaro
組織名/Organization	国立成育医療研究センター	National Center for Child Health and Development
部署名/Division name	政策科学研究部	Department of Health Policy
住所/Address	東京都世田谷区大蔵２－１０－１	Okura 2-10-1, Setagaya, Tokyo
電話/TEL	+81.3.3416.0181	
試験のホームページURL/Homepage URL		
Email/Email	rintaromori@gmail.com	

情報送信組織/Organization sending information		
送信者名/Name of person sending information	蓋若エン	Gai Ruoyan
情報送信組織/Organization	国立成育医療研究センター	National Center for Child Health and Development
所属部署/Division name	政策科学研究部	Department of Health Policy
住所/Address	東京都世田谷区大蔵２－１０－１	Okura 2-10-1, Setagaya, Tokyo
電話/TEL	+81.3.3416.0181	
Email/Email	gai-r@ncchd.go.jp	

実施責任組織/Sponsor		
機関名/Institute	独立行政法人国際協力機構(研究所)	Japan International Cooperation Agency
機関名/Institute（機関選択不可の場合）		
部署名/Department		

実施責任組織に設定する適切な機関が存在しない場合は、「その他」を選択し、「機関名（機関選択不可の場合）」に実施責任組織の機関名、及び組織名を直接入力して下さい。

研究費提供組織/Funding Source		
機関名/Organization	独立行政法人国際協力機構(研究所)	Japan International Cooperation Agency (JICA)
機関名/Organization（機関選択不可の場合）		

組織名/Division		
組織の区分/Category of Funding Organization	日本の官庁/Japanese Governmental office	
研究費拠出国/Nationality of Funding Organization	日本	Japan

研究費提供組織に設定する適切な機関が存在しない場合は、「その他」を選択し、「機関名（機関選択不可の場合）」に研究費提供組織の機関名、及び組織名を直接入力して下さい。

その他の関連組織/Other related organizations		
共同実施組織/Co-sponsor	国立成育医療研究センター	National Center for Child Health and Development
その他の研究費提供組織/Name of secondary funder(s)		

IRB等連絡先/IRBs	
倫理委員会による審査・承認/Research ethics review	あり/YES
日本の法規に定める医薬品製造業者等による医薬品の市販後調査への該当/Post marketing survey by drug manufacture etc., specified by Japanese law.	該当なし（医薬品製造業者等でない場合は、こちらを選択ください）/Not applicable (Chose this category if you are not drug manufacture etc. in Japan)
組織名1/Organization1	国立成育医療研究センター
住所1/Address1	東京都世田谷区大蔵２－１０－１
電話1/Tel1	03-3416-0181
Email1/Email1	rintaromori@gmail.com
組織名2/Organization2	
住所2/Address2	
電話2/Tel2	
Email2/Email2	
組織名3/Organization3	
住所3/Address3	
電話3/Tel3	
Email3/Email3	

他機関から発行された試験ID/Secondary IDs		
他機関から発行された試験ID/Secondary IDs	いいえ/NO	
試験ID1/Study ID_1		

ID発行機関1/Org. issuing International ID_1		
試験ID2/Study ID_2		
ID発行機関2/Org. issuing International ID_2		
治験届/IND to MHLW		

試験実施施設/Institutions	
試験実施施設名称/Institutions	

その他の管理情報/Other administrative information	
一般公開日（本登録希望日）/Date of disclosure of the study information	2018 年 05 月 31 日

試験進捗状況/Progress	
試験進捗状況/Recruitment status	参加者募集終了-試験継続中/No longer recruiting
プロトコル確定日/Date of protocol fixation	2011 年 05 月 29 日
登録・組入れ開始（予定）日/Anticipated trial start date	2011 年 07 月 01 日
フォロー終了(予定)日/Last follow-up date	2016 年 06 月 30 日
入力終了(予定)日/Date of closure to data entry	
データ固定（予定）日/Date trial data considered complete	
解析終了(予定)日/Date analysis concluded	

関連情報/Related information		
プロトコル掲載URL/URL releasing protocol		
試験結果の公開状況/Publication of results	未公表/Unpublished	
結果掲載URL/URL releasing results		
主な結果/Results		

その他関連情報/Other related information		

更新許可者/UMIN user permited to amend	
更新許可者 UMIN ID1/UMIN ID1	
更新許可者 UMIN ID2/UMIN ID2	
更新許可者 UMIN ID3/UMIN ID3	

管理情報/Management information	
UMIN試験ID/Unique ID issued by UMIN	UMIN000031789
状態/Status	登録済み/Regist
初回申請者/Applicant for provisional registration	gai-r(GAI RUOYAN)
申請日/Date of provisional registration	2018 年 03 月 19 日
登録者/Registrant	gai-r(GAI RUOYAN)
登録日時/Registered date	2018 年 03 月 19 日
最終更新者/Last modified by	gai-r(GAI RUOYAN)
最終更新日/Last modified on	2019 年 01 月 31 日

閲覧ページへのリンク/Link to view the page	
URL(日本語)	https://upload.umin.ac.jp/cgi-open-bin/ctr/ctr_view.cgi?recptno=R000036286
URL(英語)	https://upload.umin.ac.jp/cgi-open-bin/ctr_e/ctr_view.cgi?recptno=R000036286

閲覧ページ（ICDR）へのリンク/Link to view the page(ICDR)	
URL(日本語)	https://upload.umin.ac.jp/cgi-bin/ctr/ctr_view.cgi?recptno=R000036286
URL(英語)	https://upload.umin.ac.jp/cgi-bin/ctr_e/ctr_view.cgi?recptno=R000036286

研究計画書

登録日時	登録者	ファイル名

研究症例データ仕様書

登録日時	登録者	ファイル名

研究症例データ

登録日時	登録者	ファイル名

戻る

UMIN臨床試験登録システムのご使用に関するお問い合わせは、こちらのお問い合わせフォーム からお願いいたします。それ以外のお問い合わせは、こちら よりお願い致します。
