# Supplementary material for: Strengthening the community support group to improve maternal and neonatal health seeking behaviors: A cluster-randomized controlled trial in Satkhira District, Bangladesh
Source: PLoS One. 2019 Feb 28;14(2):e0212847. doi: 10.1371/journal.pone.0212847 (PMC6394907; doi:10.1371/journal.pone.0212847)
Supplement: S5 File — (PDF) [file pone.0212847.s006.pdf]

# Impact Study on Community Support Group

## 1. Background

The Government of Bangladesh is committed to achieve the Millennium Development Goals (MDGs) and has re-affirmed its obligation in different strategic papers. Reproductive health has been identified as an area of high priority in the Health, Population and Nutrition Sector Programme (HPNSDP 2011-2016) to meet the MDG goals 4 and 5. The operational plan of maternal, neonatal and child health has identified the priority areas, that includes improvement of the quality of EmOC services from all levels of hospitals, social and community mobilization, revitalization of Community Clinics (CC) and improved participation of community for CC management through establishment of community groups.

Japan has been supporting the targets and strategies of the Government of Bangladesh in achieving the Millennium Development Goals (MDGs). To this effect, the Japan International Cooperation Agency (JICA), an executing agency of Japan's Official Development Assistance (ODA), gives priority to strengthening social sector, in particular, the areas of health, medical care and primary education. As for the health sector, JICA puts importance on the improvement of the basic health conditions through focusing its efforts on the maternal and child health, and infectious diseases. JICA provides technical cooperation to these two health sector priorities, taking advantage of its own experiences and achievements in the world.

### 1.1. Safe Motherhood Promotion Project I

With the view to reducing maternal and neonatal mortality, JICA has supported the Government of Bangladesh to implement the Safe Motherhood Promotion Project (SMPP) at Norshingdi district on pilot basis from July 2006 to June 2011. The project was aimed at establishing an effective safe motherhood service delivery system to improve the availability and utilization of quality services for women during pregnancy and child birth. To achieve the objectives and outputs, the project had both facility and community level interventions. The terminal evaluation indicates that the project has achieved its purpose.

### 1.2. Safe Motherhood Promotion Project II

On successful completion of SMPP phase I and in recognition of its achievements, the Government of Bangladesh (GoB) has requested JICA to expand the SMPP project (SMPP phase II) in other districts, especially the low performing and hard to rich districts. As such, JICA has decided to expand its technical assistance to **Shatkhira** district (main intervention district). JICA supports implementation of 5S/Kaizen/TQM at selected public health facilities to improve quality of services. SMPP is also providing technical support to the Revitalization of Community Clinic (CC) Project to develop the Community Groups and Community Support Groups for operationalization and management of CC. A cluster randomized trial is also designed in collaboration with ICDDR,B under the SMPP phase II to evaluate impact of community intervention.

The SMPP phase II interventions have been designed taking the good practices identified in Narsingdi and Chowgacha models. The goal of the SMPP phase II is to improve the maternal and neonatal health status in Bangladesh. The purpose is to expand the approaches to improve maternal and neonatal health (MNH) service quality and utilization in align with Health, Population, and Nutrition Sector Development Program (HPNSDP). The project has designed its activities focusing on the following three outputs:

- Function of the coordination platform of MNH activities among stakeholders is enhanced at national level
- Process of good practices and lessons learnt extracted from the Project are disseminated in the country
- Appropriate and replicable local implementation mechanisms of maternal, neonatal and child health (MNCH) minimum package and approaches under the Upazila Health System (UHS) are defined

## 1.3. Social capital study

In collaboration with the Osaka University, Japan, SMPP has been testing the impact of Community Support Groups (CG) on social capitals in one upazila (Kalaroa) of Satkhira district. It is a cluster-randomize controlled trial with Community Clinic (CC) as cluster. In total there are 12 unions at the upazila. Out of them, one union was excluded from the study because it did not have any CC. In total there were 21 CCs in 11 upazilas. All the 21 CCs were randomized (through permuted block randomization) to allocate 10 CCs for intervention and 11 CCs for comparison. The name of the CCs by unions and their population are given in table 1.

**Table 1. Name of Unions, Community Clinics and population by areas**

| Intervention area | | | Comparison area | | |
|---|---|---|---|---|---|
| **Union** | **Community Clinic** | **Population** | **Union** | **Community Clinic** | **Population** |
| Sonabaria | Ram Krishnapur | 6,273 | Karagachi | Hatatgonj | 7,502 |
| Jugikali | Bamankali | 5,892 | Joynagar | Dhandia | 5,630 |
| Chandanpur | Boyerdanga | 8,781 | Halatola | Domdom | 8,160 |
| Dayara | Dayara | 8,456 | Jugikali | Ofapur | 5,086 |
| Dayara | Chotosolimpur | 7,516 | Jalalabad | Jalalabad | 5,358 |
| Halatola | Halatola | 8,350 | Sonabaria | Vadiali | 6,730 |
| Karalkata | Singa | 6,000 | Karalkata | Nakila | 8,879 |
| Jalalabad | Batra | 6,971 | Kusudanga | Mohammadpur | 6,810 |
| Kusudanga | Paniquria | 5,681 | Langolzara | Talkupi | 6,540 |
| Karagachi | Kakdanga | 7,223 | Chandanpur | Kadpur | 8,235 |
| | | | Chandanpur | Hijaldi | 9,327 |
| **Total:** | | **71,143** | | | **76,522** |

The intervention for the social capital study is development of CSGs by the Community Groups (CG) attached with each of the community clinics. All the intervention area CG members will be trained on how to manage the community clinics as well as to develop CSGs under them (about 3 CSGs per CC area). On the other hand, in the comparison areas the CG members will only be trained only on management of CCs but not on development of CSGs. It is, therefore, expected that the CGs in intervention areas will develop the CSGs, as per government guideline (3 CSGs per CC), by their own efforts. On the other hand, in the comparison areas the CGs will not develop the CSGs as they do not have the knowledge and skills to do this. The objectives of the social capital study is to evaluate the impact of CSG intervention on social capital.

The impact of CSGs on social capital will be measured by the following indicators:

*Individual-level (cognitive social capital)*
- Altruism: willingness to help others

- Cooperation: willingness to collaborate with others
- Risk lovingness: tendency to take risk-taking behaviours

*Community-level (structural social capital)*
- Collective action
- Community organizations
- Institutional network

This design has created an opportunity to simultaneously evaluate the impact of CSGs on limited maternal and neonatal health indicators (especially the behavioural aspects). The baseline survey is designed to collect baseline data on selected maternal and neonatal health indicators from both the areas (intervention and comparison).

## 2. Objective of baseline survey

The proposed study will be conducted under the background of the social capital study, which is a cluster randomized community trial and is being conducted at Kalaroa Upazila of Satkhira district. The current data collection will be an extension of the parent study with the aim to assess the impact of CSGs on maternal and neonatal health seeking behaviour of recently delivered women.

This study is designed to collect baseline information from both the intervention and comparison areas to understand the current situation of maternal and neonatal health seeking behaviour of recently delivered women. The specific objectives are to:

- Determine utilization of obstetric care services (such as ANC, PNC, use of skilled birth attendants for delivery, institutional delivery rate etc.)
- Assess utilization of EmOC service during obstetric complications (met need)
- Understand the current practices related to essential newborn care
- Compare the baseline information of intervention area with those of comparison area

## 3. Indicators

This randomized trial will evaluate the changes in following indicators as a consequence of development of CSGs by the CGs after controlling for all the confounding factors:

- Proportion of targeted women received 4 or more ANC by skilled providers (Community Skilled Birth Attendants [C-SBA], Family Welfare Visitor [FWV], Nurse, Doctors)
- Proportion of deliveries attended by skilled birth attendants (C-SBA, FWV, Nurse, Doctors)
- Proportion of deliveries conducted at the health facilities including CC
- Proportion of women with complications during pregnancy/delivery/postpartum period utilized EmOC services (met need)
- Proportion of targeted women received PNC within 42 days of delivery by skilled providers
- Proportion of newborn babies received Essential Newborn Care within 24 hours

## 4. Methodology:

### 4.1. Study design:

A cross-sectional study is designed to collect baseline data from both the intervention and comparison areas. Information would be collected from the target group at a single point in time to understand the status of the indicators one year prior to the survey.

## 4.2. Study population:

The target group for this study will be the women who had a live birth between 1 June 2011 and 31 May 2012 and permanently reside at intervention and comparison areas.

## 4.3. Sample size:

The ultimate goal of the study is to determine the significance of changes (between baseline and endline) in the selected indicators that may have occurred due to intervention at the intervention area compared to the comparison area. To estimate the minimum sample size to detect the changes in indicators, with 80% power and 95% confidence level, following formula has been used. A sample size of 1,420 from each of the intervention and comparison areas is required (table 1) [total sample size is 2,840]. Table 2 shows the desired minimum sample size for the study for selected indicators.

$$n = \frac{[Z_{\alpha} \sqrt{P_0Q_0} + Z_{\beta} \sqrt{P_1Q_1}]^2}{(P_0 - P_1)^2}$$

Where,

$n$ = Desired sample size for each area
$Z$ = Standard normal deviate [1.96 for 5% level of significance]
$\alpha$ = Level of significance [0.05]
$\beta$ = Type II error [0.20 and $Z_{\beta} = 0.84$]
$P_0$ = Baseline prevalence of the indicator
$Q_0$ = $(1 - P_0)$
$P_1$ = Anticipated prevalence of the indicator after intervention
$Q_1$ = $(1 - P_1)$

**Table 2: Sample size for selected indicators at 80% power and 95% confidence level**

| Sl | Indicators | Baseline prevalence§ | Anticipated endline prevalence | Desired sample size | Sample size after adjustment for design effect (1.5) |
|---|---|---|---|---|---|
| 1 | 4 or more ANC | 29% | 35% | 945 | 1,418 |
| 2 | Delivery by SBA | 38% | 45.6% | 658 | 987 |
| 3 | Delivery at institution | 33% | 39.6% | 830 | 1,245 |
| 4 | Met need | 83% | 95% | 103 | 155 |
| 5 | Proportion of women supported by community | 3% | 15% | 86 | 129 |
| 6 | PNC within 42 days of delivery | 37% | 44.4% | 688 | 1,032 |
| 7 | Proportion of women received skilled care for neonatal sickness | 59% | 71% | 245 | 368 |

§Baseline data of Satkhira district, SMPP 2

## 4.4. Sampling method and selection of sample:

Data will be collected from both the intervention and comparison areas separately. The project data indicates that total population in the intervention and comparison areas are 71,143 and 76,522 respectively (total population: 147,665) [data by CARE CDO, Kalaroa]. Given the population size, the expected number of births in a year in intervention and comparison areas would be about 1,352 and 1,454 respectively (total births: 2,806). This estimate is based on Crude Birth Rate (CBR) 19 per 1000 population, a conservative estimate from the recent BDHS 2011 findings {CBR as per BDHS is 22.6

per 1000 population}. We have considered a lower estimate, because this area (Khulna division) is a better performing area as regards to health and family planning indicators in the country.

It has been decided to include all the eligible women (census data) both in the intervention and comparison areas, because if we consider cluster sampling the required sample size would be near to the number of eligible women living in the study area.

### 4.5. Data collection tool:

This study will use a structured questionnaire to collect relevant data from the study subjects. We propose to use the modified baseline survey questionnaire of SMPP 2 to collect data as it is already field tested (see annex 1 for data collection tool).

### 4.6. Data collection method:

Once sample is selected for the study, data will be collected by a group (n=21) of trained data collectors through face to face interview using the questionnaire. The data collectors will be trained on the objectives and contents of the questionnaire including the interview technique, selection of target group etc. However, hypothesis of the study would be masked to the data collectors to avoid interviewer (information) bias. The field activities will be supervised by two/three supervisors experienced in field activity. Each of the data collectors will interview 8-10 women daily and the data will be checked by the supervisors for consistency and omissions in the evening. All the field-up questionnaires will then be sent to the headquarters through the district office for processing.

### 4.7. Data analyses plan:

Baseline data will be primarily analyzed to calculate the indicators for intervention and comparison areas separately using the descriptive statistics. Baseline socio-demographic characteristics will also be looked at for comparison between intervention and comparison areas. At this stage there is no plan to analyze the data using analytic statistics. However, difference-in-difference statistics, after controlling for potential confounding factors, will be used to see the impact of intervention on the selected indicators once the endline data is collected, tentatively after 2 years.

## 5. Timeline for the survey

The estimated time required for the study would be 3 months (December 2012 to February 2013). Field data collection would be completed by the middle of December 2012. The draft will be prepared by the end of January. Following Gantt chart shows the activities and timeline.

**Table 3. Timeframe for the study**

| Sl | Activity | Dec '12 | | | | Jan '13 | | | | Frb '13 | | | |
|---|---|---|---|---|---|---|---|---|---|---|---|---|---|
| | | W1 | W2 | W3 | W4 | W1 | W2 | W3 | W4 | W1 | W2 | W3 | W4 |
| 1 | Preparation of data collection tool | ■ | | | | | | | | | | | |
| 2 | Recruitment of data collectors | ■ | | | | | | | | | | | |
| 3 | Training of data collectors | | ■ | | | | | | | | | | |
| 4 | Field data collection (Phase II) | | | ■ | ■ | ■ | | | | | | | |
| 5 | Data entry and cleaning | | | | | ■ | | | | | | | |
| 6 | Data analysis and construction of tables | | | | | | ■ | ■ | | | | | |
| 7 | Report writing and submission of draft report | | | | | | | | ■ | ■ | | | |
| 8 | Feedback from SMPP | | | | | | | | | | ■ | | |
| 9 | Updating and submission of the final report | | | | | | | | | | | ■ | |

**Data collection plan:**

- No. of data collectors: 21 (one each for each CC)
- No. of supervisors: 3
- Travel days: 1 day
- Training for data collectors: 2 days
- Data to be collected per day: 8-10 (average 9)
- Estimated duration of data collection: 3 weeks (including training and data collection time)
- Detail data collection plan is need to be prepared

Following table shows CC wise population, estimated births (eligible women), and number of days required for data collection etc.

| | | | | | | Data collection | | |
|---|---|---|---|---|---|---|---|---|
| **Sl.No** | **Name of Union** | **Name of CC** | **Intevention/ Comparison** | **Population** | **Estimated births** | **Days required** | **Travel & training** | **Total days** |
| 2 | Chondonpur | Hizoldi | **Comparison** | 9,327 | 177 | 18 | 3 | 21 |
| 3 | Chondonpur | Kadpur | **Comparison** | 8,235 | 156 | 17 | 3 | 20 |
| 7 | Halatala | Domdom | **Comparison** | 8,160 | 155 | 16 | 3 | 19 |
| 8 | Jalalabad | Jalalabad | **Comparison** | 5,358 | 102 | 11 | 3 | 14 |
| 10 | Joynagor | Dhandia | **Comparison** | 5,630 | 107 | 11 | 3 | 14 |
| 11 | Jugikali | Ofapur | **Comparison** | 5,086 | 97 | 10 | 3 | 13 |
| 14 | Karagaci | Hotatgonj | **Comparison** | 7,502 | 143 | 15 | 3 | 18 |
| 15 | Karalkata | Nakila | **Comparison** | 8,879 | 169 | 17 | 3 | 20 |
| 18 | Kusudanga | Mohamadpur | **Comparison** | 5,075 | 96 | 10 | 3 | 13 |
| 19 | Langolzara | Tolkupi | **Comparison** | 6,540 | 124 | 13 | 3 | 16 |
| 20 | Sonabaria | Vadiali | **Comparison** | 6,730 | 128 | 13 | 3 | 16 |
| 1 | Chondonpur | Boyardanga | Intervention | 8,781 | 167 | 17 | 3 | 20 |
| 4 | Dayara | Dayara | Intervention | 8,456 | 161 | 17 | 3 | 20 |
| 5 | Dayara | Sotosalimpur | Intervention | 7,516 | 143 | 15 | 3 | 18 |
| 6 | Halatala | Halatala | Intervention | 8,350 | 159 | 16 | 3 | 19 |
| 9 | Jalalabad | Batra | Intervention | 6,971 | 132 | 14 | 3 | 17 |
| 12 | Jugikali | Bamankali | Intervention | 5,892 | 112 | 12 | 3 | 15 |
| 13 | Karagaci | Kakdanga | Intervention | 7,223 | 137 | 14 | 3 | 17 |
| 16 | Karalkata | Singa | Intervention | 6,000 | 114 | 12 | 3 | 15 |
| 17 | Kusudanga | Paniquria | Intervention | 5,681 | 108 | 11 | 3 | 14 |
| 21 | Sonabaria | Ramkrisnopur | Intervention | 6,273 | 119 | 12 | 3 | 15 |
| | **Total:** | | | **147,665** | **2,806** | | | |

## 6. Plan for the end-line survey

There will be end-line survey conducted by the SMPP in 2015 to evaluate the impact of SMPP intervention. The data and findings of this baseline survey will be compared with those of the end-line survey. It is noted that the same methodology and variables will be used for end-line survey to ensure the comparability.

Annex 1: Data collection tool

**English Version**

# Baseline Survey:
# Impact of CSG on MNH care seeking Behavior, Kalaroa, Satkhira

## Questionnaire for data collection
## Target group: Women who had a live birth during 1st September 2011 to 31st August 2012

## Safe Motherhood Promotion Project-2, JICA

## Baseline Survey: Impact of CSG on MNH Care seeking Behavior, Kalaroa, Satkhira

### Fact sheet

| Identification | |
|---|---|
| UNION ______________________ | ☐ |
| CC Name______________________ | ☐ |
| INTERVENTION AREA=1, COMPARISON AREA=2 | ☐ |
| VILLAGE______________________ | |
| RESPONDENT'S NAME:______________________ | |

| INTERVIEWER VISITS | | |
|---|---|---|
| | | |
| DATE OF INVERVIEW | | |
| INTERVIEWER'S NAME | | |
| RESULT CODE* | | ☐ |

***RESULT CODES:**

1 COMPLETED
2 NOT AT HOME
3 POSTPONED
4 REFUSED
5 RESPONDENT INCAPACITED
6 OTHERS______________
(SPECIFY)

**List of all Community Clinics**

**List of all Unions:**

| Unions | Code |
|---|---|
| Sonabaria | 1 |
| Jugikali | 2 |
| Chandanpur | 3 |
| Dayara | 4 |
| Halatola | 5 |
| Karalkata | 6 |
| Jalalabad | 7 |
| Kusudanga | 8 |
| Karagachi | 10 |
| Joynagar | 11 |
| Langolzara | 12 |

| Intervention area | | Comparison area | |
|---|---|---|---|
| **Community Clinics** | **Code** | **Community Clinics** | **Code** |
| Ram Krishnapur | 1 | Hatatgonj | 11 |
| Bamankali | 2 | Dhandia | 12 |
| Boyerdanga | 3 | Domdom | 13 |
| Dayara | 4 | Ofapur | 14 |
| Halatola | 5 | Jalalabad | 15 |
| Singa | 6 | Vadiali | 16 |
| Batra | 7 | Nakila | 17 |
| Paniquria | 8 | Mohammadpur | 18 |
| Chotosolimpur | 9 | Talkupi | 19 |
| Kakdanga | 10 | Kadpur | 20 |
| | | Hijaldi | 21 |

# Informed consent

***(Please read to respondent before asking any question for the interview)***

Assalamaliakum/Adab,

My name is ..............................…… . I am representing the Safe Motherhood Promotion Project of JICA. We are helping Ministry of Health and Family Welfare (MOHFW) of Bangladesh in improving the reproductive health status of women and neonates in Bangladesh.

We are conducting a research to improve the maternal and neonatal health services in your area. In this regard I would like to ask you some questions about your experiences during delivery and post-partum period.

There is no possible risk if you agree to participate in this interview, although some of the questions are personal. However, all the information that you will provide will be kept strictly confidential; your name will not be used and you will not be identified in any way. This interview may take approximately 20 minutes to complete. Your participation is absolutely voluntary and there is no penalty for nonparticipation. You are free to ask any question, you may refuse to be in this interview process, you may refuse to answer any question in the interview; and you may stop the interview at any point.

Do you have any questions? Do you agree to take your interview now? Yes=1 No = 2

**If no, stop here and go to the next person for interview.**

Interviewer’s Signature:______________________________ Date: ________________

**Section 1: Background Characteristics**

| No. | Questions and Filters | Coding Categories | Go to |
|---|---|---|---|
| 100. | Have you delivered a child between **1st June 2011 to 31st May 2012?** | Yes........................................................ 1<br>No.......................................................... 2 | → Terminate interview |
| 100a. | When did you deliver your last child? | ☐☐ Month ☐☐ Year | |
| 100b. | What is the name of your youngest child? | Name:________________________ | |
| 100c. | How old is your child (name)? | ☐☐ Months. ☐ Died → | **100d** |
| 100d. | At what age.......... (Baby's name) died? | ☐☐ Months | |
| 101. | How old are you? | Age (in completed years)............☐☐ | |
| 102. | What is your religion? | Islam..................................................... 1<br>Hinduism..............................................2<br>Buddhism..............................................3<br>Christianity........................................... 4<br>Other ________________................. 96<br>(Specify) | |
| 103. | Have you ever attended any school? | Yes........................................................ 1<br>No.......................................................... 2 | → **105** |
| 104. | What is the highest class you have completed? **(If none write 00 in box)** | Class.........................................☐☐. | |
| 105. | What is your main occupation? (What kind of work do you mainly do?) | Housewife (HH works) .........................01<br>NGO job............................................... 02<br>Business ...............................................03<br>Teacher.................................................04<br>Professional (Doctor/Engineer/Advocate) 05<br>Other Govt. job ...................................06<br>Agriculture works.................................07<br>Garments Worker..................................08<br>Handicrafts .......................................... 09<br>Poultry/cattle raising.............................10<br>Skilled Laborer..................................... 11<br>Unskilled Laborer.................................12<br>Student ..................................................13<br>Beggar ..................................................14<br>House help/ Maid ............................... 15<br>Other ________________..................96<br>(Specify) | |
| 106. | What is your current marital status? | Currently married.................................. 1<br>Separated.............................................. 2<br>Divorced................................................ 3<br>Widowed...............................................4 | |
| 107. | Have your husband ever attended any school? | Yes........................................................ 1<br>No..........................................................2<br>Don't know ..........................................0 | → **109** (No, Don't know) |
| 108. | What is the highest class has your husband completed? **(If none write 00 in box)** | Class.........................................☐☐ | |

| No. | Questions and Filters | Coding Categories | Go to |
|---|---|---|---|
| 109. | What is the main occupation of your husband? (What kind of work does he mainly do?) | Unemployed ........................................ 01<br>NGO job............................................... 02<br>Business ..............................................03<br>Teacher.................................................04<br>Professional (Doctor/Engineer/Advocate) 05<br>Other Govt. job ....................................06<br>Agriculture works.................................07<br>Garments Worker..................................08<br>Handicrafts .......................................... 09<br>Poultry/cattle raising.............................10<br>Skilled Laborer.................................... 11<br>Unskilled Laborer.................................12<br>Student .................................................13<br>Beggar ..................................................14<br>Military service ....................................15<br>Rickshaw/van puller ............................ 16<br>Fisherman ............................................ 17<br>Retired.................................................. 18<br>Living abroad........................................19<br>Other ____________________.............96<br>(Specify) | |
| 110. | What is your monthly family income (from all sources)? | Taka...............[ ][ ][ ][ ][ ][ ] | |
| 111. | How many living children do you have?<br><br>**If none write "0" in box.** | Total.................................................[ ]<br>Male: Female: | |

**SECTION 2: ANTENATAL CARE (ANC)**

| No. | QUESTIONS AND FILTERS | CODING CATEGORIES | Go to |
|---|---|---|---|
| 201. | Have you received any ANC during last pregnancy? | Yes....................................................... 1<br>No........................................................ 2 | <br>→ 205 |
| 202. | How many times did you receive antenatal check up during your last pregnancy? | Times.............................................[ ][ ]<br>Don't know...........................................97 | |
| 203. | Who provided ANC to you?<br><br>**(Multiple answer)** | Qualified Doctor................................ A<br>MA/SACMO.......................................B<br>Nurse.................................................. C<br>FWV....................................................D<br>Paramedic........................................... E<br>CSBA...................................................F<br>Village doctor......................................G<br>TTBA...................................................H<br>TBA/Dai...............................................I<br>Other _______________...................... X<br>(Specify) | |

| No. | QUESTIONS AND FILTERS | CODING CATEGORIES | Go to |
|---|---|---|---|
| 204. | Where did you get the ANC checkup?<br><br>**(Multiple answer)** | District/Sadar hospital.......................... A<br>MCWC................................................B<br>UHC....................................................C<br>FWC................................................... D<br>Community clinic................................ E<br>Govt. Satellite clinic.............................F<br>NGO clinic/Satellite clinic.................. G<br>Private hospital/clinic...........................H<br>Qualified doctor's chamber...................I<br>At home................................................ J<br>Non-Qualified Doctor's Chamber........ K<br>Other ____________________............X<br>(Specify) | |
| 205. | Did you experience any problem/ complication during your last pregnancy? | Yes........................................................ 1<br>No.......................................................... 2 | <br>→ 301 |
| 205a. | What type of problems/complication you faced during your last pregnancy?<br><br>**(Multiple answers)** | High fever............................................ A<br>Severe headache/ blurred vision............B<br>Swellings of hands and face..................C<br>Severe abdominal pain..........................D<br>Convulsions/fit.................................... E<br>Any amount of vaginal bleeding............F<br>Reduced movement of the baby............G<br>Other ___________________...............X<br>(Specify) | |
| 206. | Did you go to any health facility during last pregnancy complications? | Yes.........................................................1<br>No..........................................................2 | <br>→ 301 |
| 206a. | Which facility did you visit during last pregnancy complication?<br><br>**(Multiple answers)** | District/Sadar Hospital.........................A<br>MCWC................................................ B<br>UHC.....................................................C<br>FWC...................................................D<br>Community clinic................................ E<br>Govt. satellite clinic.............................. F<br>NGO clinic/satellite clinic....................G<br>Private hospital/clinic...........................H<br>Qualified Doctor's Chamber..................I<br>Pharmacy............................................. J<br>Non-Qualified Doctor's Chamber........K<br>Other ______________.......................X<br>(Specify) | |

**SECTION 3: DELIVERY CARE**

| No. | QUESTIONS AND FILTERS | CODING CATEGORIES | Go to |
|---|---|---|---|
| 301. | Who conducted your last delivery? | Qualified Doctor.......................................01<br>MA/SACMO............................................ 02<br>Nurse.........................................................03<br>FWV......................................................... 04<br>Paramedic................................................. 05<br>CSBA........................................................06<br>TTBA........................................................07<br>TBA/Dai.................................................... 08 | |

| No. | QUESTIONS AND FILTERS | CODING CATEGORIES | Go to |
|---|---|---|---|
| | | Mother In-laws/Relative............................09<br>None..........................................................10<br>Other ______________.............................96<br>(Specify) | |
| 302. | What was the mode of delivery? | Normal........................................................1<br>Cesarean section..........................................2<br>Instrumental delivery...................................3<br>Others_____________.................................6<br>(Specify) | |
| 303. | Where did you deliver your last child? | Home..........................................................01<br>District/Sadar Hospital...............................02<br>MCWC......................................................03<br>UHC..........................................................04<br>FWC..........................................................05<br>NGO clinic/hospital..................................06<br>Private clinic/hospital................................07<br>Other ______________.............................96<br>(Specify) | |
| 304. | Did you have any problem during delivery of your last baby? | Yes............................................................1<br>No..............................................................2 | → 401 |
| 304a. | What type of problem did you face during your last birth? | Excessive vaginal bleeding.........................A<br>Severe headache and blurred vision............B<br>Convulsions/fit...........................................C<br>Prolonged labour (labour pain for more than 12 hours) ..........................................D<br>Obstructed labor.........................................E<br>Mal presentation (if any other part of the baby other than the head is seen in the birth passage, like buttocks, hand, foot or cord).....................................F<br>Retained placenta (placenta is not expelled within 30 minutes after delivery of the baby)..................................G<br>Foul smelling or greenish discharge from vagina...............................................H<br>No labor pain...............................................I<br>Leaking membrane before delivery ............ J<br>Other _____________________................X<br>(Specify) | |
| 305. | Did you go to any health facility during delivery complications? | Yes............................................................1<br>No..............................................................2 | → 401 |
| 305a. | Which facility did you visit during delivery complications? | District/Sadar Hospital................................A<br>MCWC........................................................B<br>UHC...........................................................C<br>FWC...........................................................D<br>Community clinic.......................................E<br>Govt. satellite clinic....................................F<br>NGO clinic/satellite clinic...........................G<br>Private hospital/clinic.................................H | |

| No. | QUESTIONS AND FILTERS | CODING CATEGORIES | Go to |
|---|---|---|---|
| | | Qualified Doctor's Chamber........................ I<br>Pharmacy.................................................... J<br>Non-Qualified Doctor's Chamber...............K<br>Other ______________..............................X<br>(Specify) | |

**SECTION 4: POSTNATAL CARE**

| No. | QUESTIONS AND FILTERS | CODING CATEGORIES | Go to |
|---|---|---|---|
| 401. | Did you have any physical checkup within 42 days of delivery? | Yes ........................................................1<br>No..........................................................2 → | 402 |
| 401a. | Where did you have your physical checkup after the last delivery?<br><br>**(Multiple answers)** | District/Sadar hospital.......................... A<br>MCWC.................................................B<br>UHC....................................................C<br>FWC.................................................... D<br>Community clinic................................ E<br>Govt. Satellite clinic..............................F<br>NGO clinic/Satellite clinic.................. G<br>Private hospital/clinic.......................... H<br>Qualified doctor's chamber....................I<br>At home................................................ J<br>Village doctor's chamber……………..K<br>Non-Qualified Doctor's Chamber.........L<br>Other ____________________...........X<br>(Specify) | |
| 401b. | Who provided PNC to you?<br><br>**(Multiple answers)** | Qualified Doctor................................. A<br>MA/SACMO........................................B<br>Nurse................................................... C<br>FWV....................................................D<br>Paramedic............................................ E<br>CSBA...................................................F<br>Village doctor......................................G<br>TTBA.................................................. H<br>TBA/Dai...............................................I<br>Other ______________..................... X<br>(Specify) | |
| 402. | Did you have any problems/complications during postpartum period of your last delivery? | Yes.......................................................... 1<br>No........................................................... 2 → | 501 |
| 402a. | What type of problems/complications did you face during postpartum period?<br><br>**(Multiple answers)** | Fever.....................................................A<br>Foul smelling vaginal discharge............B<br>Excessive vaginal bleeding................... C<br>Severe lower abdominal pain ...............D<br>Fits/convulsion..................................... E<br>Retained placenta (placenta is not expelled within 30 minutes after delivery of the baby)..............................F<br>Other ____________________............X<br>(Specify) | |
| 403. | Did you go to any health facility during complications/problems of postpartum period? | Yes..........................................................1<br>No...........................................................2 → | 501 |
| 403a. | Which facility did you visit during problems/complications of postpartum period? | District/Sadar Hospital.........................A<br>MCWC................................................ B<br>UHC....................................................C | |

| No. | QUESTIONS AND FILTERS | CODING CATEGORIES | Go to |
|---|---|---|---|
| | **(Multiple answers)** | FWC....D<br>Community clinic....E<br>Govt. satellite clinic....F<br>NGO clinic/satellite clinic....G<br>Private hospital/clinic....H<br>Qualified Doctor's Chamber....I<br>Pharmacy....J<br>Non-Qualified Doctor's Chamber....K<br>Other ______....X<br>(Specify) | |
| 404. | Did your baby have any problems/ complications within 28 days after birth? | Yes ....1<br>No....2 | → **501** |
| 404a. | What type of problems/complications did your baby have?<br><br>**(Multiple answers)** | Lethargy....A<br>Convulsion....B<br>Jaundice (yellow colored palm and soles). C<br>Respiratory distress (difficult & fast breathing)D<br>Repeated vomiting....E<br>Skin infection....F<br>Cord infection (redness and discharge).... G<br>Poor sucking and feeding....H<br>Fever....I<br>Hypothermia (low body temperature).... J<br>Eye infection (discharge of pus from eyes)K<br>Diarrheoa....L<br>Don't know....Y<br>Other ______.... X<br>(Specify) | |
| 405. | Did you receive any treatment for your baby's problem? | Yes ....1<br>No....2 | → 501 |
| 405a. | Who treated your baby during complications? | Qualified MBBS Doctor....A<br>MA/SACMO....B<br>Nurse....C<br>FWV....D<br>Paramedic....E<br>CSBA....F<br>Village doctor....G<br>TTBA....H<br>TBA/Dai....I<br>Others ______....X<br>(Specify) | |
| 405b. | Which facility did you visit for your baby's problems/complications?<br><br>**(Multiple answers)** | District/Sadar Hospital....A<br>MCWC....B<br>UHC....C<br>FWC/USC....D<br>Community Clinic....E<br>Govt. satellite clinic....F<br>NGO clinic....G<br>NGO satellite clinic....H<br>Private hospital/clinic....I<br>Qualified Doctor's Chamber....J | |

| No. | QUESTIONS AND FILTERS | CODING CATEGORIES | Go to |
|---|---|---|---|
| | | Pharmacy……………………………K<br>Village doctor's chamber……………L<br>Other (Specify): ______________........X | |

**SECTION 5: ESSENTIAL NEWBORN CARE**

| No. | QUESTIONS AND FILTERS | CODING CATEGORIES | Go to |
|---|---|---|---|
| 501. | Was the baby dried (wiped) immediately after delivery? | Yes ......................................................1<br>No........................................................2<br>Died immediate after birth....................3<br>Don't know..........................................7 | |
| 502. | Was the baby wrapped in cloth after drying? | Yes ......................................................1<br>No........................................................2<br>Don't know..........................................7 | |
| 503. | How long after birth was the baby bathed? | Within 6 hours .....................................1<br>Within 6-24 hours................................ 2<br>Within 2-3 days....................................3<br>After 3 days .........................................4<br>Died before bath...................................5<br>Don't know..........................................7 | |
| 504. | Was anything applied to the cord | Yes ......................................................1<br>No........................................................2<br>Don't remember....................................7 | 2, 7 →505 |
| 504a. | What was applied?<br><br>**(Multiple answers)** | Antibiotic powder/ointment................ A<br>Antiseptic (Detol/Savlon/Hexasol).......B<br>Spirit/Alcohol...................................... C<br>Mustard oil.......................................... D<br>Sindur....................................................E<br>Boric powder........................................F<br>Talcum powder....................................G<br>Ash/burnt soil...................................... H<br>Coconut oil............................................I<br>Other ______________.......................X<br>(Specify)<br>Don't know..........................................Y | |
| 505. | How long after birth the baby was (name) put to breast? | Never breast fed................................... 0<br>Within 30 minutes ............................... 1<br>More than 30 minutes but within a day…2<br>More than a day....................................3<br>Don't remember....................................7 | |
| 506. | Did you give the baby colostrum (the first liquid/milk that comes out from the breast)? | Yes ......................................................1<br>No........................................................2 | |
| 507. | Did you give baby anything else to drink (such as, water, honey, mustered oil etc.) before initiation of breast feeding? | Yes ......................................................1<br>No........................................................2<br>Don't remember....................................7 | 2, 7 →509 |
| 508. | What are those?<br><br>**(Multiple answers)** | Water.................................................. A<br>Honey.................................................. B<br>Misri/Sugar water................................C<br>Mustard oil.......................................... D<br>Fruit juice.............................................E<br>Powder milk/Baby formula..................F | |

| No. | QUESTIONS AND FILTERS | CODING CATEGORIES | Go to |
|---|---|---|---|
| | | Cow milk............................................G<br>Other liquid......................................... H<br>Other ________________________........X<br>(Specify) | |
| 509. | Did you give anything else other than breast milk during the first 6 months of birth? | Yes .......................................................1<br>No.........................................................2<br>Don't remember.....................................7 | |

**Thank the mother and finish the interview**
